# Exposure to TiO_2_ Nanostructured Aerosol Induces Specific Gene Expression Profile Modifications in the Lungs of Young and Elderly Rats

**DOI:** 10.3390/nano11061466

**Published:** 2021-06-01

**Authors:** Sarah A. Valentino, Laëtitia Chézeau, Carole Seidel, Sylvie Sébillaud, Mylène Lorcin, Monique Chalansonnet, Frédéric Cosnier, Laurent Gaté

**Affiliations:** Institut National de Recherche et de Sécurité, 1 rue du Morvan, 54510 Vandoeuvre-lès-Nancy, France; sarah.valentino@inrs.fr (S.A.V.); laetitia.chezeau@hotmail.fr (L.C.); carole.seidel@inrs.fr (C.S.); sylvie.sebillaud@inrs.fr (S.S.); mylene.lorcin@inrs.fr (M.L.); monique.chalansonnet@inrs.fr (M.C.); frederic.cosnier@inrs.fr (F.C.)

**Keywords:** inhalation, titanium dioxide, lung, age, transcriptomics, rat

## Abstract

Although aging is associated with a higher risk of developing respiratory pathologies, very few studies have assessed the impact of age on the adverse effects of inhaled nanoparticles. Using conventional and transcriptomic approaches, this study aimed to compare in young (12–13-week-old) and elderly (19-month-old) fisher F344 rats the pulmonary toxicity of an inhaled nanostructured aerosol of titanium dioxide (TiO_2_). Animals were nose-only exposed to this aerosol at a concentration of 10 mg/m^3^ for 6 h per day, 5 days per week for 4 weeks. Tissues were collected immediately (D0), and 28 days after exposure (D28). A pulmonary influx of neutrophilic granulocytes was observed in exposed rats at D0, but diminished with time while remaining significant until D28. Similarly, an increased expression of several genes involved in inflammation at the two post-exposure time-points was seen. Apart from an age-specific pulmonary influx of lymphocyte, only slight differences in physio-pathological responses following TiO_2_ exposure between young and elderly animals were noticed. Conversely, marked age-related differences in gene expression profiles were observed making possible to establish lists of genes specific to each age group and post-exposure times. These results highlight different signaling pathways that were disrupted in rats according to their age.

## 1. Introduction

The rapid development of nanotechnology may lead to the exposure of workers to nanomaterials in many industrial sectors; however, the health effects of nanoparticles are still under investigation. Titanium dioxide (TiO_2_) is one of the most commonly produced nanomaterials worldwide, with multiple uses such as in personal skincare products, pharmaceuticals, food, water treatment, and construction [1,2,3]. The main route of occupational exposure to such nanomaterials is inhalation since they can become aerosolized, especially during handling processes. The pulmonary toxicity of TiO_2_ nanoparticles has been extensively investigated, and despite the fact TiO_2_ has been classified as a poorly soluble low toxicity nanoparticle [4], inhalation studies with different rodent species and TiO_2_ samples with different physical and chemical characteristics have reported pulmonary inflammation [3,5]. At fairly high doses, the induction of fibroproliferative alterations and the impairment of nanoparticle pulmonary clearance are associated with alterations of macrophage functions [6] as well as increased occurrences of pulmonary pathologies such as cancer and fibrosis [7,8,9].

Most studies on nanomaterial lung toxicity have used healthy young rodents, however the general population includes groups of individuals more susceptible to toxicant exposure such as the elderly. Lung aging is characterized by a decline in lung function with a decrease in the volume of thoracic cavity, the alteration of muscles that aid respiration, a decrease in pulmonary reserve, the loss of airway elasticity, alveolar enlargement, impaired clearance of particles through the mucociliary elevator, and the alteration of immune response [10,11].

In a previous work [12], we have showed that the nose-only inhalation exposure of young F344 rats to a TiO_2_ aerosol (10 mg/m^3^), 6 h a day, 5 days a week for 4 weeks, led to lung inflammation decreasing over time, and changed the modulation of gene expression profile after exposure. This work along with those from others demonstrate that classical toxicology approaches need to be combined with “omics” approaches in order to assess the complex interaction between nanomaterials and living organisms and potentially identify adverse outcome pathways [13,14,15,16,17,18,19,20].

In this study, using the same nanomaterial and exposure protocol, we used conventional and molecular approaches to assess the pulmonary toxicity of TiO_2_ in elderly (19-month-old) rats. Then, we compared the toxicological profile of this nanomaterial between the two age groups. Tissue samples were collected immediately and 28 days after the end of exposure in order to observe short-term effects and potential recovery following exposure. As mentioned above, combinations of conventional toxicology and “omics” methods have been performed in several studies however to our knowledge, this is the first study which combine exposure and age effect using conventional and molecular approaches.

## 2. Materials and Methods

### 2.1. In Vivo Experimental Design, Generation, and Exposure of TiO_2_ Nanostructured Aerosol

The animal experiments were performed according to European (Directive 2010/63/EC) and French (Décret n°2013-118) legislations regarding the protection of animals used for scientific purposes. This project was authorized by the French Ministry of Higher Education and Research following the advice of the regional ethical committee (Comité d’Ethique Lorrain en Matière d’Expérimentation Animale, CE n°66) (Authorization n°00692.01 from 14 October 2013).

The inhalation system which was described previously [21] is mainly composed of an aerosol generation system (rotating brush generator RBG1000 from PALAS GmbH, Karlsruhe, Germany) and inhalation towers for nose-only exposure from EMMS (Electro-Medical Measurement Systems, Bordon, UK). Animals were exposed to either filtered air (control group) or TiO_2_ nano-aerosol (Aeroxide^®^ P25, Evonik Industries AG, Essen, Germany) at a concentration of 10 mg/m^3^ (Exposed group) for 2 periods of 3 h a day, 5 days a week for 4 weeks. Filtered air was conditioned at a temperature of 22 ± 2 °C and a humidity of 55 ± 10%. The aerosol monitoring and its characterization were ensured by real-time devices (condensation particle counter, electrical low pressure impactor, aerodynamic particle sizer, scanning mobility particle sizer spectrometer, and optical light scattering dust monitor) and off-line analyses (gravimetric filter, particle size-distribution by cascade impactor, and sampling for TEM observations). The physical and chemical characteristics of TiO_2_ P25 nanoparticles and aerosol were described previously in detail [21,22]. However, their main characteristics are presented in Table 1. All animal exposures were performed with the same batch of TiO_2_ between May 2014 and March 2015.

Following exposure, male Fisher F344 rats, 12–13-week old (300–320 g)—referred to as young adults—and 19-month old (400–425 g)—referred to as the elderly group—were necropsied immediately (D0) and then 28 days (D28) after the end of exposure (*n* = 6 per group at each time point). Animals were deeply anesthetized with Pentobarbital (Ceva Santé Animale, Libourne, France) (60 mg/kg) and exsanguinated through the abdominal aorta. Tissues were collected and weighed immediately after animal death.

### 2.2. Broncho-Alveolar Lavage Fluid (BALF)

Broncho-alveolar lavage was performed by flushing the left lobe with ice-cold PBS (Phosphate Buffered Saline, pH 7.4, Invitrogen^®^, Thermo Fisher Scientific France, Illkirch-Graffenstaden, France). Then, BALF cytology and biochemistry analyses were performed. After centrifugation of BALF (5 min, 400 g, and 4 °C in Eppendorf^®^ centrifuge, Eppendorf France SAS, Montesson, France), biochemical analysis (lactate dehydrogenase activity (LDH) and protein content) was performed on the supernatant. LDH activity was measured with a Randox RX Daytona (Method LD Opt. LDH P-L; Randox Laboratories Ltd, Roissy-en-France, France) according to the manufacturer instructions. Total proteins were measured using the Bradford technique using a BioRad 96 well-format assay kit (BioRad, Marnes-la-Coquette, France) and a Bio-Tek plate reader (BIO-TEK Instruments Inc., Winuschi, VT, USA) according to the manufacturer instructions.

The cell pellet was used for total and differential cell counts. After cytocentrifugation on microscope slides, BALF cells were stained with the May–Grünwald–Giemsa method and percentages of neutrophils, macrophages, and lymphocytes were determined after counting 500 cells (Microscope Leitz Orthoplan, Leica Microsystems, Nanterre, France).

### 2.3. Histopathological Analysis

Lung right caudal lobes were slowly filled and fixed with 4% neutral buffered formaldehyde (Sigma Aldrich, Saint Quentin Fallavier, France). Following dehydration, they were embedded in paraffin (Richard-Allan Scientific ^®^, Thermo Fisher Scientific France, Illkirch-Graffenstaden, France). Then, tissue blocks were sectioned and slides were stained using hematoxylin and eosin staining technique prior to bright field microscopy histopathology analysis (Merck ^®^, Fontenay Sous Bois, France). The histopathological assessment of the lung slides was performed by board-certified anatomic pathologists.

### 2.4. Statistical Analysis for Physiological Data

Data are expressed as: median [Q1; Q3], with first (Q1) and third quartile (Q3) corresponding to 25 and 75% of the scores, respectively. A Mann and Whitney model was used (GraphPad Prism software, version 8.0, San Diego, CA, USA) for all the biological data, except transcriptomic analysis.

### 2.5. Total RNA Extraction and Purification

Following necropsy, accessory lung lobes were collected in RNA Later (Sigma Aldrich, Saint Quentin Fallavier, France) (*n* = 6 for each group and each time-point). Then they were disrupted using a gentleMACS™ Dissociator (Miltenyi Biotech, Gladbach, Germany). Total RNAs were purified using Nucleospin RNA Midi Kit^®^ (Macherey-Nagel, Hoerdt, France). All RNA samples had an A_260_/A_280_ ratio around 2 (Nanophotometer, Implen, Munich, Germany) and an RNA integrity number between 6.4 and 9.1 (Bioanalyzer, Agilent Technologies, Les Ulis, France). RNA samples were stored at −80 °C until further use.

### 2.6. Microarray Hybridization

All transcriptomic experiments were performed according to MIAME standards [23]. Experiments were performed according to manufacture instructions (Agilent Technologies, Les Ulis, France). Briefly, 100 ng of RNAs were labeled with Cyanine 3-CTP using Low Input Quick Amp Labeling kits. Labeled cRNAs were purified using the RNeasy ^®^ Plus Mini kit (Qiagen, Courtaboeuf, France) and hybridized onto Agilent Technologies SurePrint G3 Unrestricted GE 8x60K microarrays (microarrays n° G4858A for elderly rats and G4853A for young ones). Slides were washed and scanned on an Agilent G2505C microarray scanner with a 3-µm resolution. Then, data were extracted using Agilent Feature Extraction software version 12.1. Microarray data were uploaded to the NCBI Gene Expression Omnibus database [24], where they are accessible under GEO Series accession number GSE99997 and GSE145479 for the young and elderly groups respectively (http://www.ncbi.nlm.nih.gov/geo, accessed on 18 February 2020).

### 2.7. Microarray Data Analysis

**Figure 1:**
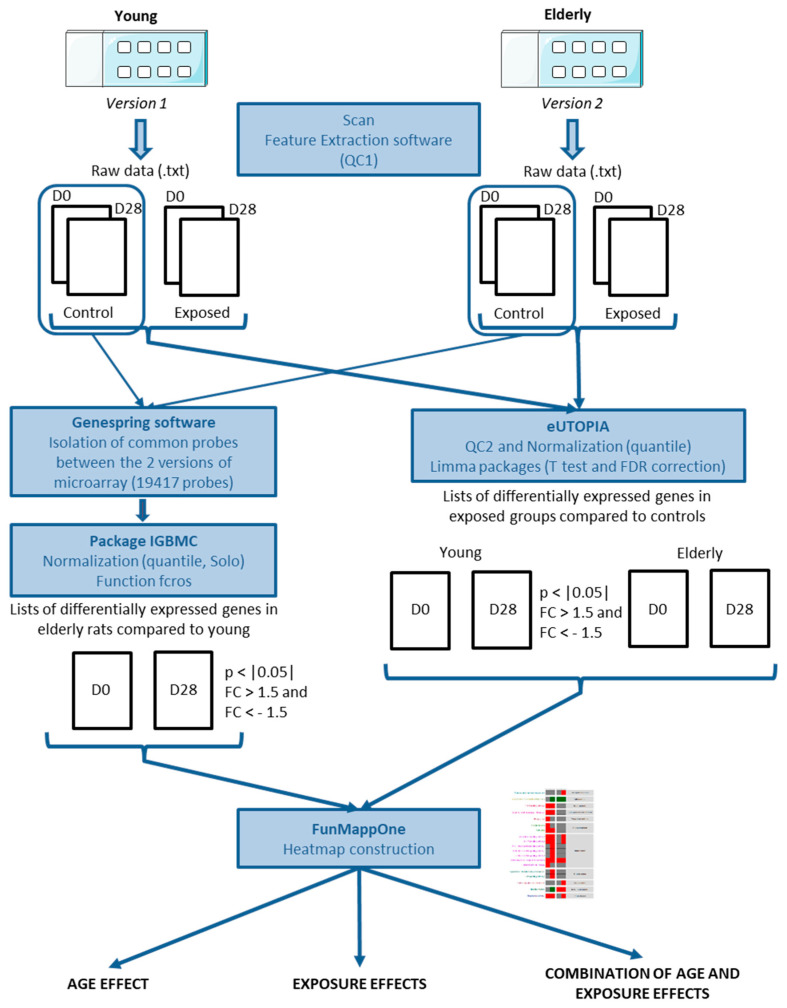
Transcriptomic analysis procedures: D0: Day 0; D28: Day 28; QC1/QC2: Quality Control 1 and 2; IGBMC: Genetic and Molecular and Cellular Biology Institute (Strasbourg, France).

Figure 1 summarizes the protocols of the transcriptomic analysis used in this work. Details are given below.

#### 2.7.1. Comparison of Gene Expression Profiles of Young and Elderly Controls

Young and elderly groups were analyzed on two different versions of rat microarray. In order to compare young and elderly control rats, we used GeneSpring software (version 14.9, Agilent Technologies, Les Ulis, France) to extract common probes between the two sets (19417 common probes). Then, Solo software was used to quantile–quantile normalize microarrays data (IGBMC, Strasbourg, http://www-microarrays.u-strasbg.fr/Solo/index.html, accessed on 5 February, 2019). For each time-point, differentially expressed genes between the young and elderly groups were identified using a method based on fold-change rank ordering statistics developed on R software (FCROS, [25]). Genes for which fold changes (FC) between elderly and young animals were at least 1.5 in either direction, and with f-values ≤ |0.05|, were considered as significantly differentially expressed.

#### 2.7.2. Comparison of Gene Expression Profiles in Young and Elderly Animals Exposed to TiO_2_ Aerosol or Filtered Air (Controls)

In order to analyze the exposure effect on the two age groups, raw data were imported in the eUTOPIA tool (solUTion for Omics data Preprocessing and Analysis, https://github.com/Greco-Lab/eUTOPIA, accessed on 10 July, 2019) [26]. Quality control and probe filtering were performed. During the quality control procedure, outliers were identified and removed if necessary. The probe filtering was quantile-based and compared the intensities of the probes to those of the negative control probes. After that, data were quantile normalized. Then, a batch correction was performed using a confounding table with all the parameters which could interfere with the biological analysis. After the annotation, a differential analysis was finally performed with the limma package using a Benjamini–Hochberg correction [27]. Genes for which the FC for exposed vs. matched controls was at least 1.5 in either direction and with *p*-values < |0.05|, were considered as significantly differentially expressed.

## 3. Results

Results concerning the pulmonary effects of the inhaled aerosol in young rats were published previously by our laboratory [12]. In addition, gene expression profiles from young animals freely available from the GEO database were analyzed using the same protocol as for the elderly rats in order to study similarities and differences between the two age groups.

### 3.1. Biometry

There is a significant increase of body weight due to age and growth between control groups (Fold Change, FC = 1.23 and FC = 1.17, *p* = 0.0022 and *p* = 0.0022 at D0, day of the end of exposure, and D28, respectively; Table 2).

In the young group, exposure to TiO_2_ nano-aerosol induced a slight decrease in bodyweight at D0 (FC = 0.94, *p* = 0.0152) and an increase in lung weight to bodyweight ratios (FC = 1.26, *p* = 0.0022). Twenty-eight days after the end of exposure, there was no difference in bodyweight between control and exposed animals, but exposure to TiO_2_ induced a decrease in lung weight to bodyweight ratio (FC = 0.88, *p* = 0.0087). In the elderly group, following exposure to TiO_2_ nanoparticles, there was no change in bodyweight (Table 2). Lung weight to body weight ratio was significantly increased at D0 in the exposed group compared to the control one (FC = 1.10, *p* = 0.0303; Table 2) but no such difference was seen at D28.

### 3.2. BALF Cytology and Biochemistry

In the control groups, a significant increase in neutrophilic granulocyte influx was observed in elderly rats in comparison to young rats, in term of percentage of total cells (Table 2). At the end of exposure (D0), the percentage of neutrophilic granulocytes in BALF was strongly and significantly increased in both exposed groups compared to control (FC = 31.9, *p* = 0.0079 and FC = 8.4, *p* = 0.0095 for young and elderly rats, respectively; Table 2). This neutrophil influx decreased with time but remained significantly higher in exposed groups in comparison to controls (FC = 6.9, *p* = 0.0190 and FC = 3.3, *p* = 0.0079 for young and elderly rats, respectively, at D28). While no significant change in lymphocytes was seen in young rats following TiO_2_ exposure, in elderly rats a significant increase of these leukocytes was observed at D0 and at D28, (*p* = 0.0043 and, *p* = 0.0238, respectively; Table 2).

In the control groups, age induced a slight increase in LDH activity at day 0 for the elderly group compared to the young one (FC = 1.77, *p* = 0.0390). There was, however, no effect of age on protein concentration in BALF. After exposure to TiO_2_ aerosol, LDH activity was significantly increased up to day 28 in both exposed groups as compared to concurrent controls (FC = 4.14, *p* = 0.0022 for young and FC = 2.11, *p* = 0.0173 for elderly at day 0 and FC = 1.52, *p* = 0.0043 for young and FC = 1.59, *p* = 0.0159 for elderly at day 28). In both exposed groups, protein concentration was significantly increased at day 0 (FC = 1.65, *p* = 0.0087 for young and FC = 1.71, *p* = 0.0216 for elderly at day 0) but no difference was seen at day 28.

### 3.3. Lung Histopathological Analysis

**Figure 2:**
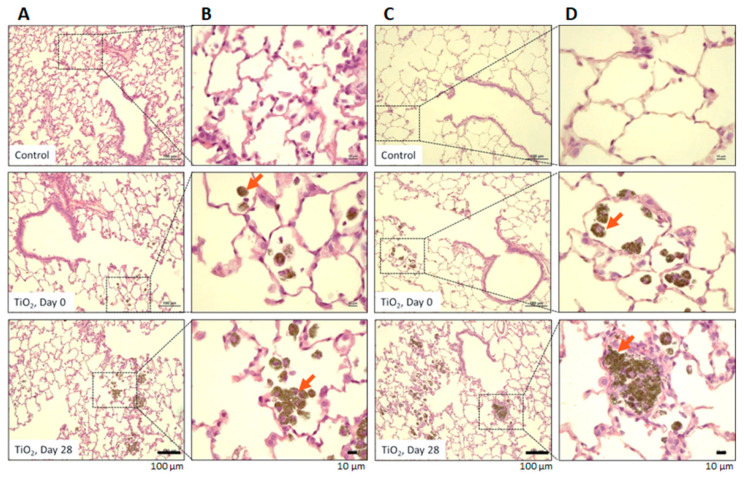
Lung Histopathology: Representative microscopy images of hematoxylin-eosine stained lung sections of controls and exposed to TiO_2_ aerosol of both age. (**A**,**B**) are from lungs of young control and exposed rats, (**C**,**D**) are from lungs of elderly control and exposed rats. Red arrows indicate macrophages containing nanoparticles. (**A**,**C**) magnification ×40; (**B**,**D**) magnification ×200.

Exposure to TiO_2_ led to similar histopathological changes in the lungs of young and elderly animals (Figure 2). The description of histopathological modifications seen in young rats have been published previously [12]. For elderly animals, on D0, several scattered particle-laden macrophages and a few small aggregates of macrophages were found in the alveolar lumen. In addition, particles were noted at the level of the cilia of epithelial cells and in the lumen of bronchioli and bronchi, associated with very few particle-laden macrophages. Minimal and multifocal alveolar epithelialization was seen in alveolar walls, corresponding to the proliferation of Type II pneumocytes. On D28, a few scattered particle-laden macrophages and several large aggregates of macrophages were also observed in the alveolar lumen, particularly next to alveolar ducts and terminal bronchioli. In addition, a few particle-laden macrophages were seen on the epithelial surface of bronchioli and bronchi. Minimal, multifocal alveolar epithelialization was noted around most of the particle-laden macrophage aggregates. The presence of nanoparticles into macrophages was thoroughly investigated by looking at BALF cells using optical and electronic microscopy (Supplementary Figure S1).

### 3.4. Transcriptomic Analysis

#### 3.4.1. Age Effect

**Figure 3:**
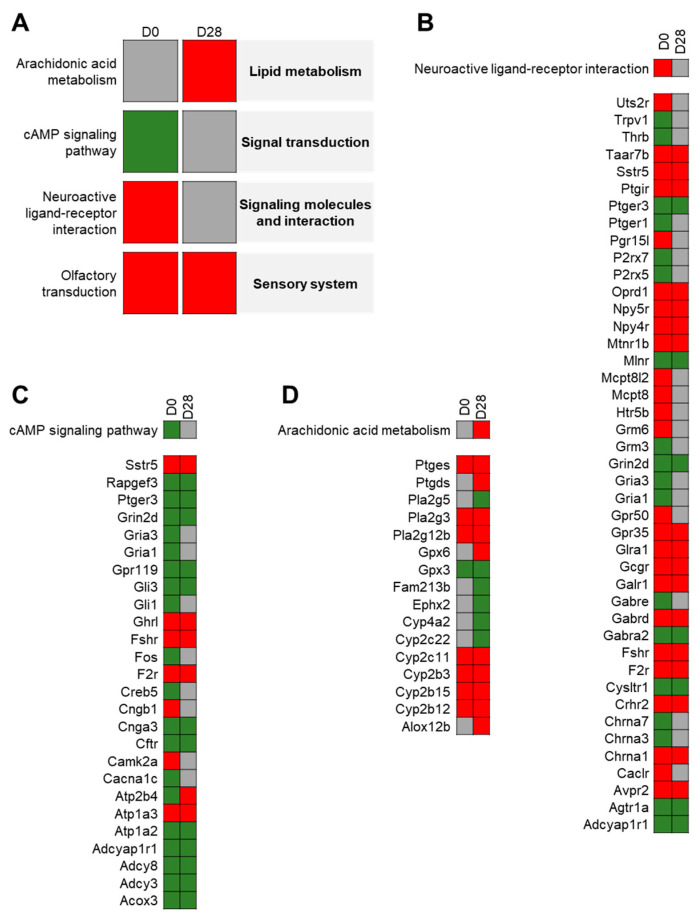
Age effect on control rats: Heatmap from the FunMappOne tool. Global disturbing pathways (**A**) and detailed genes for “*cAMP signaling pathway*” (**B**), “*Neuroactive ligand-receptor interaction*” (**C**) and “*Arachidonic acid metabolism*” (**D**) were deregulated in elderly control rats compared to young control group. The first column represents the data for day 0 (D0) and the second for day 28 (D28). The color of the boxes represents the level of expression of a majority of genes involved in this pathway: green for under-expression in elderly animals compared to young ones, red for over-expression, and grey for no difference.

On D0, in the control groups, the analysis of lung samples from control groups using FCROS software revealed that 1223 genes were differentially expressed (DEGs) between young and elderly animals with 647 under-expressed genes and 576 over-expressed genes in the lungs of elderly rats compared to young rats (Figure 3A). With FunMappOne tool and the KEGG database, deregulated biological pathways with mainly over-expressed genes were involved in “*Neuroactive ligand-receptor interaction*” (Figure 3C) and “*Olfactory transduction*”. The “*cAMP signaling pathway*” (Figure 3B) was also deregulated but with mainly under-expressed genes.

On D28, 1328 DEGs have been observed in lungs of elderly control rats compared to young control ones, with 639 under-expressed genes and 689 over-expressed genes. Two pathways were deregulated and composed of a majority of over-expressed genes, “*Arachidonic acid metabolism*” (Figure 3D) and “*Olfactory transduction*”.

#### 3.4.2. Exposure Effect on the Young Group

**Figure 4:**
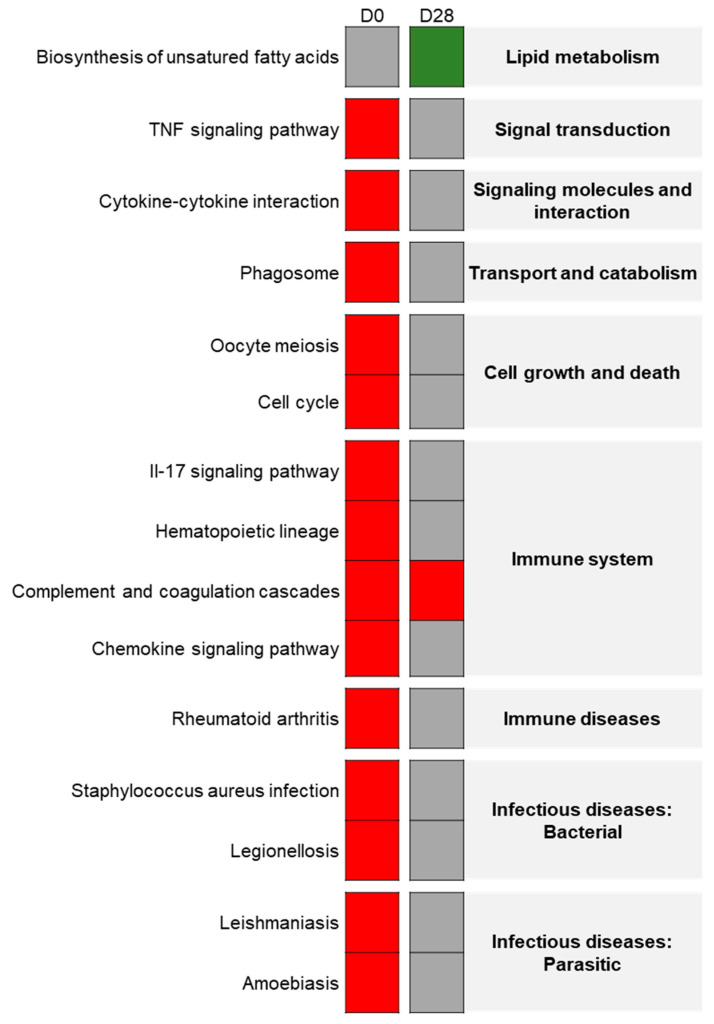
Exposure effect on young rats: Heatmap from the FunMappOne tool. The first column represents the data for day 0 (D0) and the second for day 28 (D28). The color of the boxes represents the level of expression of a majority of genes involved in this pathway: green for under-expression in exposed group compared to controls, red for over-expression, and grey for no difference.

Using the eUTOPIA tool to reanalyze the dataset of the young rat group from Chézeau et al. [12], 572 DEGs were found on D0 in the lungs of the exposed group, with 173 under-expressed genes and 399 over-expressed ones. Using the FunMappOne tool and the KEGG database, most of the deregulated biological pathways, with mainly over-expressed genes, were shown to be involved in inflammatory and immune responses such as “*TNF signaling*”, “*Cytokine-Cytokine receptor interaction*”, “*Phagosome*”, “*IL-17 signaling pathway*”, “*Chemokine signaling pathway*” and “*Complement and coagulation cascades*” (Figure 4).

On D28, only 128 DEGs were found in exposed animals, among which 37 were under-expressed and 91 over-expressed. The most deregulated biological pathway mainly associated with over-expressed genes was “*Complement and coagulation cascades*” (Figure 4).

#### 3.4.3. Exposure Effect on the Elderly Group

**Figure 5:**
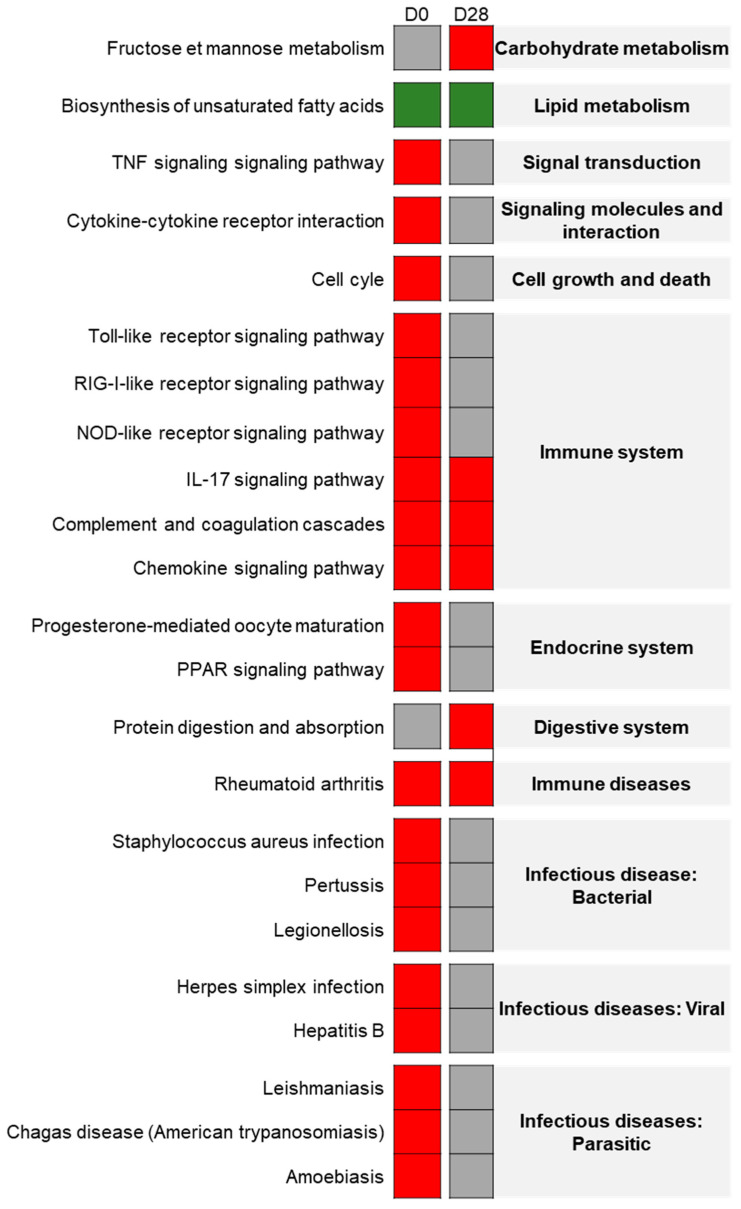
Exposure effect on elderly rats: Heatmap from the FunMappOne tool. The first column represents the data for D0 and the second for D28. The color of the boxes represents the expression of a majority of the genes involved in this pathway: green for under-expression in exposed group compared to controls, red for over-expression, and grey for no difference.

On D0, in elderly rat lungs, 612 DEGs were identified with 262 genes under-expressed and 350 over-expressed. Using the FunMappOne tool and the KEGG database, most of the deregulated pathways, with mainly over-expressed genes, were shown to be involved in inflammatory and immune response pathways such as “*Cytokine-Cytokine receptor interaction*”, “*TNF signaling pathway*”, “*IL-17 signaling pathway*”, “*Chemokine signaling pathway*”, and “*Complement and coagulation cascades*” (Figure 5). Also, there were different pathways involved in infectious and immune diseases where over-expressed genes were the same as in inflammatory pathways. On the other hand, “Biosynthesis of unsaturated fatty acids” was mainly composed of under-expressed genes (Figure 5).

Twenty-eight days after the end of exposure, 472 DEGs were observed with 143 genes under-expressed and 329 over-expressed. Using the KEGG database, some pathways appeared to be deregulated in a similar way to the one on day 0 like “*IL-17 signaling pathway*”, “*Complement and coagulation cascades*”, and “*Chemokine signaling pathway*” (Figure 5). Two new deregulated pathways were involved in “*Fructose and mannose metabolism*” and “*Protein digestion and absorption*” (Figure 5).

#### 3.4.4. Combination between Age and Exposure to TiO_2_ Aerosols

**Figure 6:**
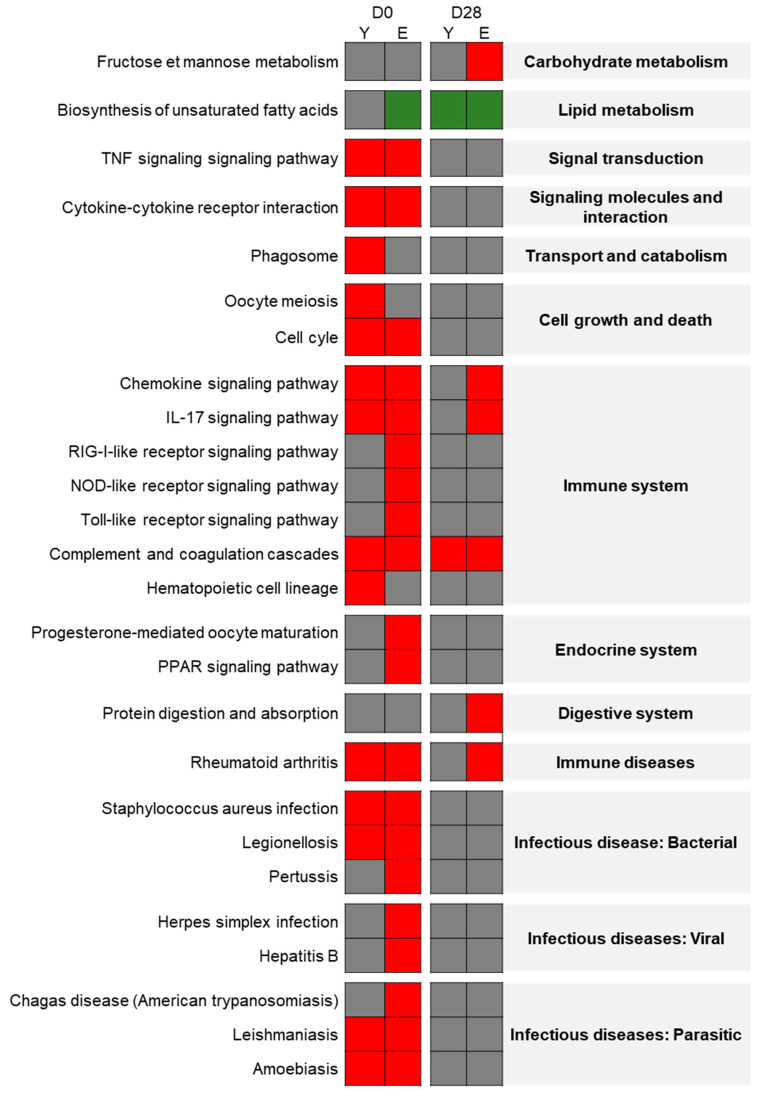
Age effect on exposed rats: Heatmap from FunMappOne tool. Columns 1 and 2 represent the data for D0 and columns 3 and 4 for D28. Columns 1 and 3 represent the data from the young rat group (Y) and columns 2 and 4 from the elderly rat group (E). The color of the boxes represents the expression of a majority of the genes involved in this pathway: green for under-expression, red for over-expression, and grey for no difference.

**Figure 7:**
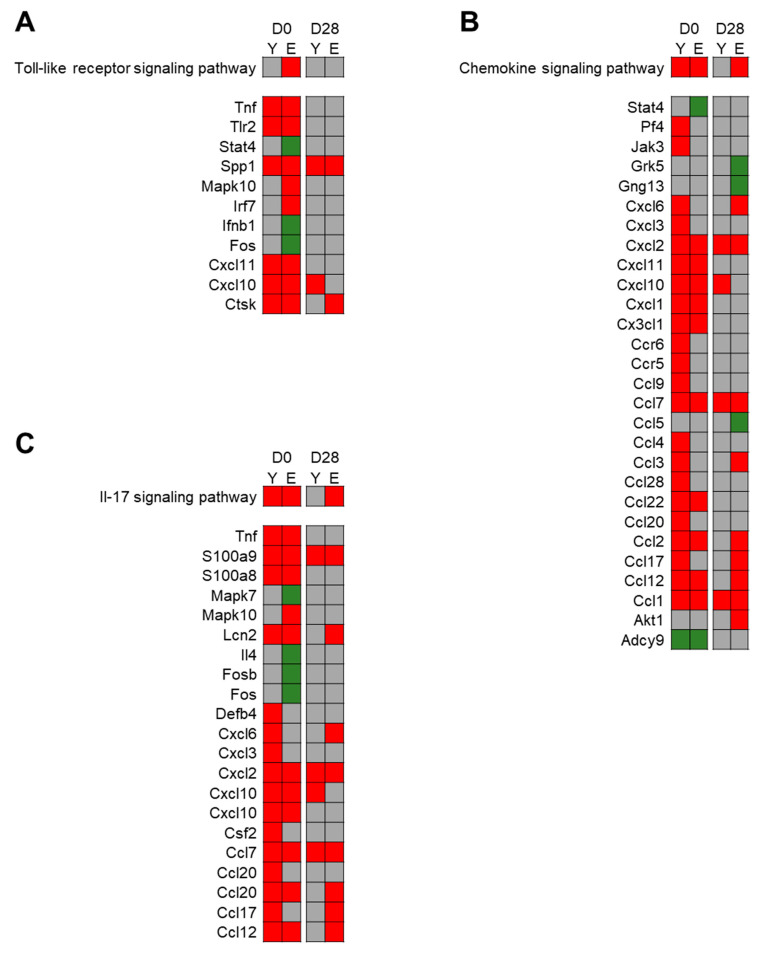
Age effect on exposed rats—Detailed deregulated pathways: Heatmap from FunMappOne tool. Detailed DEGs were presented for “*Toll-like receptor signaling pathway*” (**A**), “*Chemokine signaling pathway*” (**B**) and “*Il-17 signaling pathway*” (**C**). “Columns 1 and 2 represent the data for day 0 (D0) and columns 3 and 4 for day 28 (D28). Columns 1 and 3 represent the data from the young rat group (Y) and columns 2 and 4 from the elderly rat group (E). The color of the boxes represents the expression of the genes involved in these three pathways: green for under-expression, red for over-expression, and grey for no difference.

Table 3 summarizes the number of DEGs in the three conditions (common to both exposed groups or specific to young or elderly exposed rats) and at each time-point. The full list of DEGs could be found in Supplementary Table S1. Figure 6 and Figure 7 present the heatmaps for the analysis of the age effect on the exposed groups.

##### DEGs Common to Young and Elderly Rats

On D0, 230 DEGs were common to young and elderly exposed rats with 35 under-expressed genes and 195 over-expressed ones. Most of the deregulated pathways, with mainly over-expressed genes, were involved in inflammatory response, like “*TNF signaling pathway*”, “*Cytokine-Cytokine receptor interaction*”, “*Chemokine signaling pathway*”, “*IL-17 signaling pathway*”, and “*Complement and coagulation cascades*” (Figure 6). Five pathways involved in infectious and immune diseases were deregulated with common DEGs with inflammatory pathways.

On D28, 90 DEGs were common between young and elderly exposed rats, among which 23 were under-expressed and 67 over-expressed. One pathway, composed of over-expressed genes, was deregulated and involved in “*Complement and coagulation cascades*”. Only genes involved in “*Biosynthesis of unsaturated fatty acids*” pathway were under-expressed in both groups at this time-point (Figure 6).

##### Specific DEGs in Young Rats

On D0, 342 DEGs were found to be specific to young exposed rats with 138 genes under-expressed and 204 over-expressed (Table 3). Two inflammatory pathways appeared to be deregulated: “*Phagosome*” and “*Hematopoietic cell lineage*” (Figure 6).

Twenty-eight days after the end of exposure, 38 DEGs were detected in the lung tissue of which 14 were under-expressed and 24 over-expressed; however, no pathway appeared to be deregulated.

##### Specific DEGs in Elderly Rats

On D0, 382 DEGs specific to elderly rats were found with 227 under-expressed genes and 155 over-expressed genes. One pathway mainly included under-expressed genes: “*Biosynthesis of unsaturated fatty acids*”. Three pathways involved in inflammatory response appeared to be specifically deregulated in elderly exposed rats with “*RIG-I-like receptor signaling pathway*”, “*NOD-like receptor signaling pathway*”, and “*Toll-like receptor signaling pathway*” (Figure 7A). Four pathways involved in infectious diseases were also deregulated, but the DEGs involved in them were shared with inflammatory pathways.

On D28, 382 DEGs were observed only in the elderly exposed lungs with 120 under-expressed genes and 262 over-expressed genes. “*Fructose and mannose metabolism*”, “*Chemokine signaling pathway*” (Figure 7B), “*IL-17 signaling pathway*” (Figure 7C), and “*Protein digestion and absorption*” pathways were deregulated, with most DEGs over-expressed.

## 4. Discussion

This study brings new insights into the impact of age on the pulmonary effects of repeated nanoparticle exposures through conventional and molecular toxicological approaches.

### 4.1. Comparison of Experimental Conditions of Exposures to TiO_2_ Nano-Aerosols

In this study, although the experiments were not performed at the same time, young and elderly animals were exposed following the same protocol 6 h per day, 5 days per week for 4 weeks to 10 mg/m^3^ of the same nanostructured aerosol of TiO_2_ P25. As described previously [12], taking into account the interspecies difference in terms of respiratory parameters and estimated pulmonary deposited fractions, the cumulated deposited dose normalized to lung alveolar cell surface area following animal exposure was approximately the same than that following the daily occupational exposure of a worker at 0.3 mg/m^3^ (the NIOSH recommended exposure limit for ultrafine TiO_2_ [28]) throughout part of his career: 35 h per week, 45 weeks per year for about 20 years.

Due to similar respiratory minute volume [29] and similar clearance, cumulative masses of titanium deposited in lungs at the end of the exposure period and lung burdens over the recovery period were comparable in all exposed young and elderly animals [22]. This lack of difference in terms of experimental conditions and the absence of dose-related bias made possible the direct comparison of the pulmonary toxicological properties of such an aerosol in the two age groups and then to assess whether aging could affect the physio-pathological response in rodents.

Concerning the selected time-points, day 0 allowed evaluating the acute pulmonary response immediately after the end of exposure and day 28 to better understand the potential recovery mechanisms put in place after the end of treatment. Despite the fact that we were able to assess the long-term toxicological effects up to 180 days after the end of exposure for young adults, as shown by Chézeau et al. (2018), we were unable to pursue our investigations beyond 28 days post-exposure with elderly rats due to the lack of healthy animals.

### 4.2. Age Effect

In our study, we observed only minor physiological difference between the two control groups. Neutrophil and lymphocyte percentages were slightly higher in the control elderly group compared to the control young one. These results may be associated with what has been reported in the literature. Indeed, aging usually induces pulmonary changes in innate and adaptive immunity, with decreased phagocytic function of macrophages, reduced activity of natural killer cells, altered serum levels of pro-inflammatory cytokines, higher number of airway neutrophils, and decreased T-cell stimulation by dendritic cells [30,31,32].

Age-related effects have been studied from physiological, histological, and molecular angles in the context of various diseases like lung cancer, chronic obstructive pulmonary disease, and asthma. However, the molecular consequences of aging alone on lungs has been given little attention so far. Our work shed new light on this aspect. Indeed, we obtained a specific age-related gene expression profile. For instance, “*cAMP signaling pathway*” was found to be down-regulated. This observation could be compared to that of Vanscheeuwijck et al. who showed that forskolin stimulation of cAMP synthesis was decreased in elderly (24–25 months) with regard to young (2–3 months) Wistar rats [33]. In our study, “*Olfactory transduction*” pathway was also disturbed with mainly over-expressed genes. Kolbe and collaborators found that olfactory receptors were expressed in airway smooth muscle cells and modulate physiological processes as contractility and hyperproduction of inflammatory protein [34]. In “*Arachidonic acid metabolism*” pathway, Cyp2C was over-expressed that may suggest an increase in epoxyeicosatrienoic acids production, which are anti-inflammatory mediators [35]. This “inflamm-aging” status was described by Lowery et al. with an increase in production of cytokines and a deregulation of the balance pro- and anti-inflammatory mediators [11].

### 4.3. Combination of Age and Exposure Effects—Short-Term Response

Immediately after the end of exposure to TiO_2_ nano-aerosol, levels of induced lung inflammation were comparable in young and elderly rats. Most studies focusing on pulmonary response to TiO_2_ exposure in young adult rodents also highlighted lung inflammation and changes in biochemical parameters [5,15,20,36,37]. However, the most striking difference between the two age groups was the increase of lymphocytes in elderly animals only.

This difference in the cytological BALF profile could be linked to the specific gene expression profile observed in the elderly exposed rats, especially with three up-regulated pathways associated with leukocyte regulation: “*RIG-I-like receptor signalling*”, “*NOD-like receptor signaling*”, and “*Toll-like receptor signalling*” pathways. For each of them, the deregulated genes were involved in activating the production of pro-inflammatory cytokines and chemokines. For the “*Toll-like receptor signaling*” pathway, the deregulated genes would lead to “*Chemotactic effects of T cells*”. Toll-like receptors are key molecules in the recognition and initiation of the innate immune responses, and stimulate a range of inflammatory cytokines like the IL-6 family and TNFα [11,38]. TLR2, which was over-expressed in our case, is expressed by dendritic cells and on the surface of activated and memory T cells [39]. Its function as a costimulatory receptor molecule for T cell activation helps to maintain T cell memory [40]. In the “*Cytokine-cytokine receptor interaction*” pathway, some genes appeared to be elderly-specific: *IL-4*, *Ifnb1*, and *Ackr4* were under-expressed and *Tnfsf9*, *Tnfrsf12a*, *IL-11*, and *Gdf3* were over-expressed. IL-4 is an inhibitory cytokine produced by Th2 cells and inhibits inflammatory cytokine production by macrophages [41,42]. *Ifnb1* transcription is repressed at the late phase of macrophage activation [43]. These dysregulations could lead to an activation of interaction with the TNF/TGFβ and IL-6/IL-12-like families and an inhibition of interaction with Cxcl-type chemokines and IL-4 families. IL-6, a pleiotropic cytokine, is involved in immunity mechanisms, by promoting the Th17 differentiation and the proliferation of cytotoxic T cells [38]. IL-11, which was over-expressed in our study, is also a pleiotropic cytokine of the IL-6 cytokine family, that plays a key role in the remodelling of the airways and the development of airway hyper-responsiveness [44].

### 4.4. Combination of Age and Exposure Effects—Recovery Response

Twenty-eight days after the end of exposure, less severe but still significant lung inflammation was observed in both age groups but without any major difference between young and elderly animals.

At this stage, although no physio-pathological difference was noted, the molecular approach highlighted an elderly-specific gene expression profile with the up-regulation of *Cxcl2*, *Ccl7*, and *Ccl1* and the dysregulation of the “*Chemokine signaling*” pathway, including down-regulated (*Grk5*, *Gng13*, and *Ccl5*) and over-expressed (*Cxcl6*, *Ccl3*, *Ccl2*, *Ccl17*, *Ccl12*, and *Akt1*) genes. Using the KEGG database, our results lead to the hypothesis of the activation of the Akt pathway with the down-regulation of Grk5 and Gng13 and the up-regulation of different cytokines and chemokines. The activation of the Akt pathway could lead to cytokine production, cell survival, migration, apoptosis and cellular growth, and differentiation. Xie et al. showed that the PI3K/Akt pathway was involved in pro-inflammatory production [45]. The “*IL-17 signaling*” pathway was also up-regulated with elderly-specific over-expression of *Lcn2*, *Cxcl6*, *Ccl2*, *Ccl17*, and *Ccl12* genes. The activation of NF-κB signalling alters the interaction of IL-17 with receptors, which could lead to the activation of autoimmune pathology, neutrophilic recruitment, and immunity to extracellular pathogens. Since a more sustained induction of inflammation-related pathways by TiO_2_ nano-aerosol in elderly animals is described one month after the end of exposure, one may wonder whether this process may be observable at longer post-exposure time-points. Even-though we were not able to verify this due to the lack of animals, from a physio-pathological viewpoint, persistent inflammatory response may contribute to more pronounced adverse effects and pathologies in the elderly population than in younger individuals.

## 5. Conclusions

Our study provides new insights into the effect of age on the pulmonary toxicity of inhaled TiO_2_ nano-aerosol using conventional and molecular approaches. Despite the fact that there was little difference in terms of inflammation and histopathological changes between the two age groups, except for an increase in lymphocytes in elderly exposed animals immediately after the end of exposure, striking variances in terms of gene expression profiles between young and elderly rats, even without any treatment, were observed. Our data demonstrate that there is a need to further investigate the effect of age on the toxicity of nanomaterials and ultrafine environmental nanoparticles to better understand why older populations have a different susceptibility to such exposure. Molecular approaches such as transcriptomics appear to be efficient tools for achieving this goal.

## Figures and Tables

**Table 1:** Main titanium dioxide particle and aerosol characteristics.

Particle Size (nm)	Specific Surface Area (m^2^/g)	Aerosol Mass Concentration (mg/m^3^)	Aerosol Number Concentration (Particle/cm^3^)	CMAD (nm) (ELPI)	MMAD (nm) (SIOUTAS)	Ti Lung Deposited Dose (mg/Lung) *
21.5 ± 7.2	51	10.17 ± 3.29 (young)	24,000 ± 6400	269 (GSD: 2.22)	905 (GSD: 2.19)	2.08 ± 0.09
		10.42 ± 1.80 (elderly)				2.19 ± 0.40

CMAD: Count Median Aerodynamic Diameter; MMAD: Mass Median Aerodynamic Diameter; GSD: Geometric Standard Deviation. Full particle and aerosol characteristics have already been published [21]. * Titanium lung deposited doses at Day 0 were from Gaté et al. [22].

**Table 2:** Physiological parameters.

Parameters		Time-Point	Young Rats				Elderly Rats			
			Control	Exposed	Exposure Effect (*p*-Value and Fold Change)		Control	Exposed	Exposure Effect (*p*-Value and Fold Change)	
Biometry	Body weight (g)	D0	296.0 [289.0; 302.3]	278.5 [267.8; 288.5]	*	0.94	364.0 [357.0; 380.5]	358.0 [349.5; 360.5]	NS	0.98
		D28	341.1 [333.0; 345.4]	352.7 [342.9; 359.4]	NS	1.03	414.5 [384.5; 430.3]	425.0 [394.5; 447.5]	NS	1.03
	Lung weight to body weight ratio	D0	0.0042 [0.0039; 0.0044]	0.0053 [0.0051; 0.0056]	**	1.26	0.0049 [0.0046; 0.0051]	0.0054 [0.0053; 0.0057]	*	1.10
		D28	0.0049 [0.0046; 0.0052]	0.0043 [0.0041; 0.0045]	**	0.88	0.0044 [0.0042; 0.0050]	0.0045 [0.0044; 0.0048]	NS	1.02
BALF cytology	Neutrophils percentage (%)	D0	1.60 [1.20; 2.00]	51.0 [50.0; 57.0]	**	31.90	5.400 [3.700; 7.250]	45.20 [36.80; 49.20]	**	8.37
		D28	2.10 [1.95; 3.45]	14.5 [11.2; 20.8]	*	6.90	7.600 [5.000; 7.800]	25.40 [19.20; 28.00]	**	3.34
	Lymphocytes percentage (%)	D0	0.20 [0.20; 0.40]	0.00 [0.00; 0.20]	NS	/	0.600 [0.400; 1.100]	4.200 [4.000; 4.800]	**	7.00
		D28	0.00 [0.00; 0.05]	0.300 [0.20; 0.40]	NS	/	0.000 [0.000; 0.000]	0.800 [0.400; 1.200]	*	/

Lung biometry and BALF cytology are presented for young and elderly rats (*n* = 6 per group). All data are presented as median [Q1; Q3] and *p*-value are presented as NS = no significance, * *p* < 0.05 and ** *p* < 0.01. Data for young rats were reanalyzed from Chézeau et al. [12].

**Table 3:** Number of DEGs on day 0 and day 28 after exposure in both groups.

	Day 0			Day 28		
	Total	↘	↗	Total	↘	↗
Common to both exposed groups	230	35	195	90	23	67
Specific to young exposed rats	342	138	204	38	14	24
Specific to elderly exposed rats	382	227	155	382	120	262

Number of total DEGs (FC ≥ |1.5| and f-value ≤ |0.05|) at the two time-points (day 0 and day 28) that were commonly or specifically deregulated in young and/or elderly rats. **↘**: Downregulated genes; ↗: Upregulated genes.

## Data Availability

Microarray data have been deposited in the Gene Expression Omnibus database where they are accessible under GEO Series accession number GSE99997 and GSE145479 for the young and elderly groups respectively (http://www.ncbi.nlm.nih.gov/geo, accessed on 18 February 2020). Other data are available upon request.

## Supplementary Materials

The following are available online at https://www.mdpi.com/article/10.3390/nano11061466/s1, Figure S1: Representative images of BALF and lung cells, Table S1: Differentially expressed genes following TiO_2_ exposure.
