# Supplementary material for: Exposure to TiO2 Nanostructured Aerosol Induces Specific Gene Expression Profile Modifications in the Lungs of Young and Elderly Rats
Source: Nanomaterials (Basel). 2021 Jun 1;11(6):1466. doi: 10.3390/nano11061466 (PMC8230065; doi:10.3390/nano11061466)
Supplement: Supplementary file 1 [file nanomaterials-11-01466-s001.zip › nanomaterials-1198273-supplementary.pdf]

Exposure to TiO_2 Nanostructured Aerosol Induces Specific Gene Expression Profile Modifications in the Lungs of Young and Elderly Rats

Sarah A. Valentino, Laëtitia Chézeau, Carole Seidel, Sylvie Sébillaud, Mylène Lorcin, Monique Chalansonnet, Frédéric Cosnier and Laurent Gaté *

Institut National de Recherche et de Sécurité, 1 rue du Morvan, 54510 Vandoeuvre-lès-Nancy, France;
sarah.valentino@inrs.fr (S.A.V.); laetitia.chezeau@hotmail.fr (L.C.); carole.seidel@inrs.fr (C.S.);
sylvie.sebillaud@inrs.fr (S.S.); mylene.lorcin@inrs.fr (M.L.); monique.chalansonnet@inrs.fr (M.C.);
frederic.cosnier@inrs.fr (F.C.)

* Correspondence: laurent.gate@inrs.fr; Tel.: +33-(0)383508504

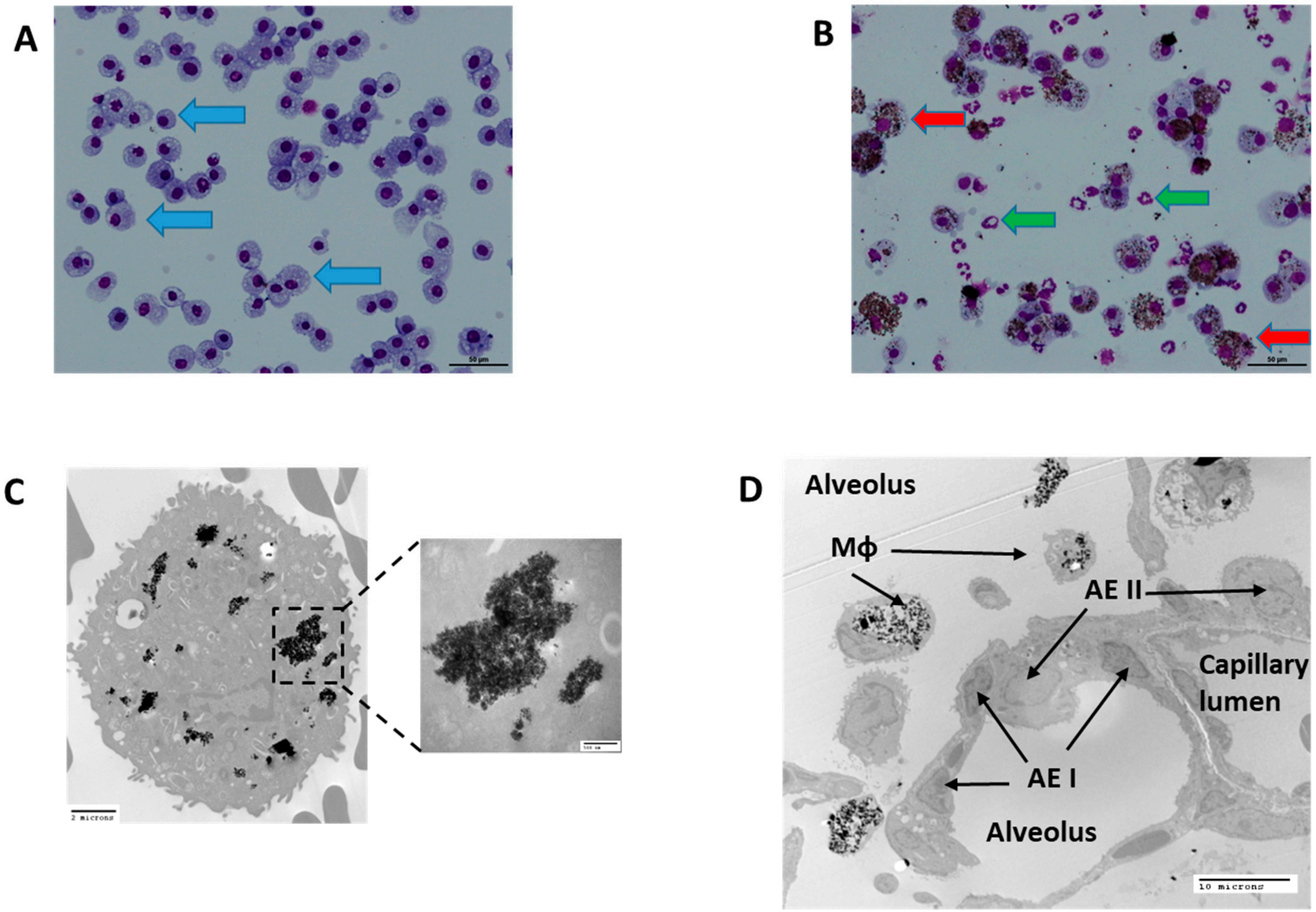

Figure S1: Representative images of BALF and lung cells. A) Representative image of BALF cells from control animals (D0) stained with MGG method. B) Representative image of BALF macrophages from exposed animals to TiO_2 (D0) stained with MGG method. C) Representative image of Transmission Electron Microscopy of a BALF macrophage from animals exposed to TiO2 (D0). Blue arrow: macrophage; Red arrow particle-ladden macrophages; Green arrow: Neutrophilic granulocytes. D) Representative image of Transmission Electron Microscopy of lung tissue from animals exposed to TiO2 (D0). AE I: Type I Alveolar epithelial cell; AE II: Type II Alveolar epithelial cell; Mφ: Macrophage.

Table S1: Differentially expressed genes following TiO_2 exposure.

DEGs in exposed young rats						DEGs in exposed elderly rats					
Day 0			Day 28			Day 0			Day 28		
Gene Symbol	Adjusted p-value	FC	Gene Symbol	Adjusted p-value	FC	Gene Symbol	Adjusted p-value	FC	Gene Symbol	Adjusted p-value	FC
LOC108348108	3.42192E-05	0.143610602	Zbtb16	0.00059379	0.22390785	LOC689840	6.07252E-21	0.182064282	Scd	1.23575E-14	0.180684417
Nr1d1	4.87333E-11	0.149384661	Scd	4.9884E-07	0.30447483	Scd	9.02646E-14	0.245136514	LOC683600	8.00845E-07	0.278771519
Dbp	9.51173E-08	0.251672011	LOC681458	7.9839E-07	0.37378474	Nr1d1	5.39289E-10	0.253069743	LOC681458	1.5466E-13	0.307903302
Cyp2a3	6.37714E-08	0.372778813	Arntl	2.5241E-07	0.40622165	LOC102551157	7.01661E-19	0.263534651	Scd4	4.68066E-14	0.314465342
Rnase3	3.29682E-12	0.382886372	Hs3st6	1.5772E-06	0.40802007	Cacng5	7.96852E-20	0.264499351	Fcrla	6.18073E-07	0.352106101
Ccdc116	3.99871E-05	0.390449586	Slc6a2	2.0354E-05	0.42481056	Mepe	1.39424E-12	0.284186766	Cyp26b1	4.57451E-11	0.404710375
Nr1d2	1.49585E-10	0.432043906	Scd4	4.0779E-07	0.43588298	Tent5b	7.28003E-12	0.302930617	Arntl	2.41368E-08	0.410851804
Gpihbp1	5.78347E-10	0.437864383	Fibin	4.5632E-08	0.45035248	Olfm2	1.55302E-20	0.304076269	Lpal2	2.1322E-07	0.446586635
Dll1	1.45548E-06	0.450144542	Fam13a	1.252E-07	0.45937838	LOC102554475	1.90618E-19	0.309614417	RGD1566401	2.16383E-06	0.45047256
Amigo2	2.69072E-06	0.453454269	Cry1	4.6691E-08	0.46393402	Klf15	1.96676E-14	0.318019509	Rnase3	1.06438E-10	0.456430914
Cyp26b1	0.000921229	0.45918988	Zp1	9.8577E-05	0.46775108	Ccdc116	3.06021E-10	0.322939818	Npr3	1.94699E-12	0.459687221
Klf15	4.65834E-06	0.459951691	Npr3	7.8791E-06	0.49030446	LOC102555924	1.93481E-16	0.329963563	Hs3st6	1.61955E-09	0.467349878
Npr3	5.11741E-07	0.466978415	Spon2	0.00012061	0.49824694	LOC102546772	7.91159E-23	0.330810612	Igsf21	2.82354E-10	0.47570738
Nrarp	1.96012E-06	0.474171999	Fkbp5	0.00694255	0.51118125	Cep295nl	2.69584E-15	0.332446583	LOC685321	1.24524E-09	0.481552447
Rprml	7.97388E-05	0.47630782	Eln	1.906E-05	0.54017355	Zbtb16	4.47664E-10	0.333841	Eln	3.63533E-06	0.482097166
Mlc1	5.21591E-06	0.47674829	Adamts9	1.8908E-06	0.54025948	LOC103691910	9.93753E-17	0.342092517	Rflnb	9.04868E-13	0.493140278
Fmo3	1.4201E-05	0.483945428	Kcnmb2	3.0287E-11	0.54914088	Ifnb1	6.27695E-18	0.349149005	Kcnj3	1.29418E-11	0.496019514
Tac1	1.94896E-10	0.484854312	Wfdc1	2.7273E-08	0.56619249	Tmem106c	6.27695E-18	0.350974433	Bpifa1	1.12604E-05	0.504653764
Acer2	1.63186E-07	0.493753396	Adm	6.0348E-05	0.57010602	LOC102554817	1.26781E-16	0.362566544	Sgcg	1.0006E-11	0.509010366
Sirpb3	0.002645333	0.498911598	Rflnb	2.5241E-07	0.59273455	Mogat1	1.34921E-19	0.36345698	Fam13a	1.40276E-11	0.50915649

Slc6a2	6.3956E-05	0.502521546	Cyp4b1	5.4481E-09	0.60267051	Bcl2l10	2.63989E-18	0.365612263	Cry1	1.11527E-15	0.510973188
Dusp1	0.006829516	0.505611265	Rtp3	2.2329E-05	0.60377847	Cyp2a3	5.61165E-11	0.380223905	Prf1	0.000601224	0.51213661
Tent5b	0.000782916	0.507079098	Lims2	0.00025609	0.62387084	Lca5l	2.59318E-18	0.380692477	LOC102556873	3.15475E-10	0.517849105
Tef	1.36901E-08	0.508144427	Acsm5	2.2218E-05	0.6283611	LOC103691374	3.59533E-16	0.382379613	Rnase17	8.69043E-10	0.527001857
Dnajb1	8.50698E-06	0.508334368	Ly49s3	2.7932E-05	0.63592086	Per1	1.36626E-08	0.383518277	LOC102552044	1.37276E-14	0.541840606
Rasd2	9.01435E-06	0.514639815	Ephx4	9.7831E-08	0.64061786	Zp1	3.92457E-11	0.387955596	Hamp	3.78651E-07	0.544882308
Tesc	5.35064E-08	0.515863489	LOC689064	0.00085218	0.6419608	LOC100363510	1.20032E-06	0.410244288	Zp1	1.01961E-07	0.544986748
Zp1	0.00012515	0.519476563	Npas2	0.00028547	0.64422749	Il4	4.04631E-16	0.415588464	Fibin	2.92951E-10	0.546622761
Olr1395	2.90484E-07	0.523811233	Medag	0.00063879	0.64623365	Serpinb10	8.07548E-10	0.417674588	Ass1	1.35352E-14	0.5540887
Ciart	0.001364779	0.532617683	Abca8	1.366E-06	0.64799167	LOC681458	1.89527E-11	0.417960379	Hspb1	4.19701E-13	0.555989951
Marc1	4.50725E-09	0.533116677	Rnase3	1.2583E-05	0.65012835	Dbp	0.000100202	0.420662613	Clca1	1.14203E-08	0.556902553
Hspb1	1.29502E-09	0.533816512	Hsph1	0.00233771	0.65081999	Zfp282	1.01896E-11	0.422217486	Sema7a	6.8653E-09	0.558787382
Upk1a	1.11444E-08	0.53426778	Plxna2	1.8937E-05	0.65385899	LOC103691782	1.66875E-15	0.424785612	Fpr2	3.36081E-06	0.558853924
Plat	4.62288E-09	0.534570654	Hspb1	5.2037E-06	0.65608431	LOC102551811	5.76423E-14	0.428131327	Gng13	1.14085E-08	0.560118759
Gnat1	1.0155E-08	0.535983233	Acer2	0.00085234	0.66449843	Gzmc	0.00610089	0.43001888	Foxc1	3.81193E-10	0.560980667
RGD1308117	5.65787E-07	0.540178496	Tac1	1.2368E-05	0.66475682	Scd4	8.02601E-12	0.433900212	Ephx4	8.61806E-11	0.564093237
Dnaja1	0.000396288	0.544656766	Alas2	0.00205738	0.66719304	Rprml	8.91393E-11	0.437026148	Cbs	0.000141407	0.56965674
Actg2	1.29506E-06	0.547155023	Slc6a12	4.6338E-06	1.49688236	LOC102551408	1.27781E-17	0.437551146	LOC102550790	1.53322E-07	0.573663679
Alox15	1.04139E-10	0.547737327	Dusp2	3.7719E-05	1.49911403	Lpal2	1.76125E-07	0.445071474	LOC103692235	6.9989E-05	0.573912541
Cldn5	2.91045E-10	0.551636934	Entpd3	8.353E-08	1.50054573	Mageb7	7.3332E-13	0.448036137	Gimap4	7.78733E-16	0.574260276
Klf4	2.22561E-05	0.5545234	Cldn10	5.2943E-10	1.50165677	Sidt1	2.41967E-16	0.457576178	Asic4	3.31794E-09	0.576084505
Dlk1	1.29753E-05	0.560334607	Cdkn2a	4.1361E-09	1.50693451	Mep1a	1.55302E-20	0.461800761	Prkce	8.85219E-12	0.57663707
Sostdc1	1.44113E-05	0.560720994	LOC102552326	1.1443E-05	1.507515	Cct6b	4.21498E-10	0.463001194	Wfdc1	4.1789E-10	0.57702422
Dusp8	0.000319495	0.564318188	Grin2c	3.8025E-07	1.50803237	LOC100911163	0.00865951	0.469603977	Cyp4b1	5.14389E-13	0.579244903
LOC685184	4.50725E-09	0.564438641	Fetub	1.5856E-09	1.5189314	Spon2	3.03358E-12	0.473762587	Alox15	1.93055E-08	0.582726819
Epm2a	4.03272E-06	0.568425703	Slamf9	2.7145E-05	1.52198394	LOC690813	4.62711E-09	0.483823004	Slc34a3	3.52928E-09	0.583725996
Abcb1b	1.61758E-06	0.569577697	Itprid1	2.228E-07	1.52692363	Scart1	8.51298E-09	0.489264734	Zfp365	1.0134E-12	0.584515455
Kcnj3	1.19924E-08	0.570330516	Jchain	0.00139526	1.52727324	Esm1	0.001782339	0.491901271	Dydc2	5.28612E-09	0.585436196
Snai1	0.00010461	0.570723513	Ceacam4	4.5066E-05	1.52843934	LOC102556026	1.14938E-13	0.50003795	Npas2	7.22053E-07	0.587388306

Avil	2.18451E-09	0.57158246	Adora2b	0.00012592	1.52864534
Nat8f5	8.53421E-07	0.571969496	Tagap	2.3245E-05	1.53040593
Cyp2e1	8.79844E-06	0.572304287	Bhlhe41	2.5241E-07	1.53192155
Cplx1	2.36834E-05	0.573203787	Prss30	3.8805E-05	1.53224635
Alpk3	3.17441E-07	0.573455019	C4bpa	1.1513E-08	1.53298941
Ndrg2	3.4533E-08	0.574090509	Sct	8.789E-05	1.5347308
Ephx4	3.29852E-10	0.57521885	Tmem86a	2.2055E-05	1.5412692
Lyve1	3.03511E-08	0.576307959	Gckr	1.6539E-08	1.54346762
Ahsa2	1.42447E-05	0.577001399	Shcbp1	0.00024884	1.5471471
Klf2	0.005934855	0.577037428	Vsig2	0.00051717	1.54884701
Adamts9	2.04952E-06	0.581940801	Kcnj13	9.55E-06	1.56103755
Fn3k	1.30825E-07	0.582565129	Klk13	3.2405E-07	1.56164298
Lims2	1.04961E-05	0.583140508	Prkar1b	9.4879E-08	1.56858089
Wfdc1	9.70021E-09	0.585438111	Atp6v0d2	0.00065076	1.57398109
Asb11	0.005075624	0.588149838	Styk1	0.00245525	1.57725383
Lcat	5.12151E-08	0.588441014	RGD1563231	0.00791518	1.58370069
Atp1a2	5.22512E-08	0.590448831	Slc7a11	3.4458E-08	1.59041026
Cytl1	6.93957E-07	0.593106861	Rasl10a	6.9873E-07	1.59111812
Adhfe1	3.03052E-09	0.594120693	Epm2a	0.00020723	1.59727077
Cspg4	1.91892E-06	0.594220691	Ccne1	8.852E-06	1.60259145
Pthlh	2.09861E-06	0.594436561	Tac4	6.9072E-08	1.60694501
Aoc3	3.24633E-10	0.596058822	Sel1l3	2.1677E-09	1.62700983
Gpr4	2.11336E-08	0.596424512	Tmprss9	1.3022E-07	1.63193639
Igfbp2	4.07969E-10	0.597536934	Mt1	2.6223E-08	1.6358
Foxf1	7.58716E-06	0.597912926	Lilrb4	6.9072E-08	1.64021281
Egfl7	3.86658E-08	0.59810047	Tmprss4	7.1799E-08	1.64998266
Adamts1	0.000152734	0.603504627	Tcap	0.00474102	1.66939509
Tns2	1.12233E-09	0.604417517	LOC102552128	1.4209E-07	1.69075866
LOC690319	6.61259E-08	0.605226408	Ptx3	7.5912E-10	1.69268631

LOC102554140	2.02966E-13	0.500311057	LOC102554932	2.15608E-06	0.587551558
Agrp	4.54668E-07	0.501480284	Fgfbp1	1.12692E-08	0.587904166
LOC103692211	1.11911E-10	0.501705779	Gzma	0.00149304	0.58933122
Olr428	2.59318E-18	0.502671981	Sell	1.26963E-08	0.590364457
Map3k6	3.3104E-11	0.503951998	Hba-a2	3.42722E-06	0.592182531
Hamp	5.05171E-08	0.504496981	Vom2r15	1.29985E-06	0.599435496
Sypl2	2.95011E-09	0.505012154	Ube2ql1	1.32623E-12	0.59962087
LOC100910802	1.45343E-13	0.506023742	Gzmm	0.000179158	0.603084075
LOC102547784	5.94399E-15	0.507647499	Ihh	2.53473E-09	0.605232767
LOC100912233	5.76181E-18	0.509591335	Olr309	1.0265E-07	0.606535734
Dnase2b	1.35072E-08	0.514198496	Fmo1	3.34123E-06	0.609495308
Gadl1	0.000137394	0.516915163	Edn3	1.06438E-10	0.609827513
LOC685321	7.22193E-09	0.520801484	Cd93	4.68066E-14	0.611197861
Prrt4	3.1592E-12	0.522984016	Scd2	8.56743E-10	0.611230348
Slc34a3	1.10819E-10	0.524533847	Ppp1r16b	2.61302E-13	0.611968241
LOC102547437	1.29151E-11	0.528790585	Atp1a3	5.14389E-13	0.614282292
Adamts1	2.34E-14	0.530629621	Dlk1	6.44741E-07	0.614549361
Tcf21	3.01159E-11	0.535880369	Vtn	6.01186E-09	0.619018728
LOC498222	1.89468E-08	0.537588694	Rtp3	5.83385E-09	0.621425146
RGD1562431	7.59822E-16	0.539223635	Krt19	5.49175E-12	0.621497761
LOC103694103	4.12672E-13	0.539913064	C1qtnf6	9.5975E-07	0.621870861
Galnt14	1.05918E-06	0.540089436	Ecm1	1.09835E-09	0.622565265
Ephx4	1.59917E-11	0.540632721	Id4	2.63775E-09	0.623764338
Cyp4b1	2.32367E-14	0.540667369	Chrng	3.3411E-08	0.624569388
Slc25a29	6.75957E-13	0.541535607	Nkg7	0.005578269	0.625444911
Olr1606	1.21746E-14	0.541997996	Igfbp2	1.12886E-06	0.626329363
Rnase17	1.55118E-09	0.542836695	Tpbgl	3.88382E-10	0.628026793
Abra	9.4837E-11	0.544091958	Treml4	8.72188E-13	0.628214357
Bpifa1	4.94263E-05	0.544344672	Sh2d1a	0.008276188	0.628449649

P4ha2	4.12829E-10	0.606948504	Chst1	1.4209E-07	1.6964472
Esm1	8.66284E-06	0.607616801	Col9a2	1.1124E-08	1.69694129
Serpinb10	3.44428E-06	0.608642882	Per2	0.00300303	1.70261016
Cdr2	6.48589E-09	0.609478535	Car5a	2.9663E-06	1.70695813
Omd	3.37268E-05	0.609757436	Fcrl2	1.0056E-05	1.71315974
Anxa3	1.72312E-08	0.610155442	Tmem45b	1.6514E-06	1.71316166
Per2	0.001978728	0.610315313	Duox1	1.9293E-06	1.72960637
Per3	0.000170123	0.610532837	Itih1	1.2746E-09	1.73089626
Gnat2	1.09687E-08	0.611456757	Per3	0.00014418	1.76778523
Ly49s3	1.74436E-06	0.612942348	Tef	1.2378E-06	1.77623875
Dysf	1.79769E-07	0.613505068	Rbm3	7.7562E-08	1.78320919
Nos3	7.62061E-09	0.613914826	Bhlha15	1.8759E-06	1.78406412
LOC685827	3.30496E-07	0.614798869	Rhbdl2	7.2656E-06	1.78996944
Mapt	1.33515E-07	0.615231714	Itgam	8.5667E-07	1.79085457
Tinagl1	6.87242E-09	0.616106085	LOC100302465	2.1276E-07	1.83436103
Kcnmb4	0.00012826	0.617141133	Prmt8	6.2263E-06	1.8738658
Cyp2f4	4.50725E-09	0.617315383	C3	1.8484E-09	1.89006277
Map3k6	0.000150443	0.617321211	Hp	5.2943E-10	1.8968943
Rnase17	1.29753E-05	0.617358404	S100a9	6.7624E-05	1.94771156
LOC102554371	0.004802859	0.619794906	Gsg1	5.7092E-05	1.97815931
Mctp1	4.313E-08	0.620004566	Acot1	0.0007296	1.98028698
Rftn2	3.59748E-09	0.621695816	Nr1d2	3.1616E-08	2.00427925
Tnxb	2.54328E-08	0.622874338	Cxadrl1	1.4802E-09	2.02465965
Tnxa-ps1	9.11707E-08	0.623025648	Slc26a4	1.6252E-06	2.04977898
Notch4	1.13981E-08	0.624467693	Mylk2	3.762E-10	2.05104374
Otud7a	0.000498357	0.624526305	Chia	9.5308E-11	2.10931028
Procr	9.55289E-07	0.625661496	Fstl4	3.2704E-11	2.16749098
Ly49i3	9.94986E-06	0.626072544	Scn10a	3.7034E-09	2.24216247
Slc7a13	9.65853E-06	0.626114846	RGD1309110	6.1044E-09	2.25494552

Dusp1	3.17955E-06	0.544774972	LOC103691073	2.15299E-07	0.628493027
Tmcc1	8.62007E-13	0.545193003	Slc6a4	3.94547E-07	0.628719302
Hgd	1.23424E-12	0.546012281	RGD2301395	0.000663722	0.629673639
RGD1566401	8.2756E-05	0.547233519	LOC103692025	2.64756E-13	0.629921935
Cox8b	9.89179E-11	0.548247604	Fpr2l	1.1591E-08	0.631420667
Mapk7	6.25154E-14	0.551643813	Ndufa4l2	6.59469E-08	0.633723366
Ear1	1.86134E-11	0.551908499	Gatm	3.53174E-11	0.633816179
LOC103692821	1.04293E-07	0.554299105	Sox18	7.11654E-06	0.634102035
Tead1	1.6578E-13	0.555735559	Hsph1	2.95344E-10	0.635392213
Pde6g	1.18393E-16	0.559829618	Sec14l1	2.02767E-10	0.637936357
Dcn	2.08606E-11	0.561706636	Plppr3	1.99133E-14	0.638286441
Tril	5.76181E-18	0.565019992	Slc29a1	1.59917E-09	0.639072246
Lamc3	7.17206E-14	0.566054568	Nrarp	2.90225E-07	0.639443445
Slc2a10	1.81477E-07	0.567846078	Fam183a	6.50984E-09	0.639661061
Slc7a10	5.09852E-14	0.569171299	Pdzd2	6.94223E-10	0.640258752
Klf9	1.06793E-10	0.574693272	LOC103694228	0.00392277	0.640349716
Gstm7	5.41139E-12	0.57489644	Tek	2.66241E-08	0.640593769
Nipal1	4.58919E-07	0.57604369	Foxe1	4.42629E-07	0.641613283
Themis	9.63161E-10	0.577408821	Cald1	2.02227E-10	0.641816725
Acer2	5.02357E-08	0.580802421	Rasl11a	1.5466E-13	0.642084859
Tcap	3.23606E-08	0.581295205	Klrb1a	0.003304136	0.642601096
Hmx2	6.1381E-14	0.583433159	Adgrl2	5.14561E-11	0.643608519
Igfbpl1	1.98153E-10	0.586535001	Myl7	2.02348E-11	0.643978772
Tspyl4	5.01979E-11	0.587322235	Akap12	8.32927E-11	0.644407333
Sparcl1	1.0967E-10	0.58973582	Ndufaf2	5.30428E-06	0.645975647
LOC103692544	3.67715E-16	0.589848761	Wscd1	2.12541E-11	0.647162857
Cd19	2.07781E-08	0.590125897	Ccdc39	1.84369E-11	0.648874906
Vom2r15	7.76026E-07	0.590732675	Mtus2	1.69977E-06	0.650384803
LOC103691073	2.92443E-08	0.593684507	Nrp1	2.64181E-10	0.650693171

Cyp4b1	3.03052E-09	0.627916795	Cfi	6.6599E-11	2.2552324
Slc38a5	2.59788E-08	0.628632685	Chi3l1	7.097E-12	2.27346978
Tmcc2	5.90967E-08	0.629299584	Ccdc116	0.00071725	2.27793262
Ccdc198	3.86447E-07	0.629829903	Defb5	7.3525E-09	2.27806347
Fbxo10	9.74147E-07	0.630212023	Bpifb1	7.1769E-08	2.32454088
Scd	0.0063299	0.631221397	Rasd2	1.0642E-06	2.40145395
Cd300lg	1.8981E-06	0.632591616	Mmp7	2.1282E-07	2.41710537
Sult5a1	1.92414E-07	0.632751221	Ccl1	7.2068E-08	2.4939991
Eng	1.01072E-08	0.6331543	Cd177	5.9909E-08	2.50013817
Hsph1	0.000429239	0.635089864	Zar1	0.00979357	2.6953508
Ascl2	4.91958E-10	0.635593614	Aqp3	2.9407E-06	2.82055277
Cped1	6.61634E-07	0.636446911	LOC362795	0.00048536	2.87430287
Mfap5	2.34676E-11	0.636938664	Muc3	3.0234E-10	3.07272285
Smad6	1.46792E-07	0.638021016	Kng1	6.6599E-11	3.46332313
Vom2r15	0.000278226	0.638539588	Spp1	3.4209E-06	3.64011763
Akr1c19	5.89364E-10	0.63869949	Kng2	1.2868E-12	4.53101941
Hyal2	5.17143E-05	0.639062924	Orm1	1.5495E-11	5.75631579
Bcl6b	0.000110233	0.640332859	Nr1d1	1.0191E-09	6.0915127
Tuba3a	3.90774E-06	0.641430313	Retnla	3.0287E-11	6.19501227
Cbs	3.89382E-05	0.642018455	Lpo	1.7624E-11	6.46352819
Sun5	4.38543E-05	0.642047493	Dbp	2.8073E-10	9.07625236
Adcy9	1.09548E-07	0.642542792			
Hspb8	1.98923E-08	0.643955917			
Ppl	4.06588E-07	0.644473924			
Tbx6	9.87938E-06	0.644644203			
Cdo1	0.000177538	0.645847105			
Klf9	6.29656E-05	0.64630267			
Ctrl	8.33255E-06	0.647538558			
Rapgef3	8.24212E-08	0.647847776			

Wif1	1.39547E-09	0.594488875	Apln	3.47877E-10	0.651191696
Kcnmb2	1.85594E-09	0.595160302	Antxr1	1.42026E-10	0.651300687
Cyp24a1	7.58014E-11	0.596248083	Olfml2b	0.00595994	0.651635524
Pim3	2.13095E-09	0.5970984	Gucy1a2	7.97848E-11	0.653645575
Neb	4.24479E-09	0.597645549	LOC102549751	5.02239E-11	0.653757718
Tnxa-ps1	9.81569E-09	0.59831645	Cpne5	3.15693E-05	0.653830298
Myh6	6.6237E-10	0.598773067	Fbp1	1.1585E-08	0.653831379
LOC102556289	5.8509E-15	0.6001381	Ehd4	4.4617E-10	0.654087565
LOC102548169	6.75957E-13	0.601571509	RGD1311575	2.05456E-06	0.654864123
Col6a4	4.82207E-08	0.602223237	Csrp3	5.81095E-10	0.655071344
Rgs11	9.50615E-10	0.603078466	Grk5	4.48925E-10	0.656502787
Abca9	1.79318E-13	0.605000321	LOC102550604	1.92494E-05	0.657326527
Tpcn2	0.000279435	0.60514956	Coro2b	5.37493E-11	0.657522287
Omd	5.69486E-14	0.605543511	Kcnmb2	7.92248E-08	0.657526759
Sema6d	1.02811E-06	0.606160942	LOC103690933	5.93085E-09	0.658236082
LOC102550275	4.98303E-11	0.606814574	Hspb2	1.27171E-10	0.659251976
LOC103694306	7.48141E-17	0.607232127	Hoxb6	1.36656E-10	0.659575097
Fiz1	8.32824E-10	0.60793576	Scgb3a1	3.80155E-10	0.660192732
Phactr1	2.351E-08	0.608494071	LOC103691157	1.73751E-08	0.660219208
Fam107a	2.51309E-07	0.608811738	LOC102550720	3.98198E-09	0.660219826
LOC102546975	3.13117E-10	0.609223557	Gfra2	8.8247E-13	0.660659564
LOC102557105	2.72087E-07	0.609970586	Cdkl5	8.24381E-09	0.661442248
Scd2	6.21004E-10	0.610206453	Hspb3	5.33195E-12	0.661684045
Gp2	2.46088E-10	0.610371438	Exph5	2.10661E-10	0.662865495
Igsf21	2.29538E-07	0.610921157	Alas2	7.72985E-05	0.662868101
LOC103691621	1.47161E-11	0.611779181	Zbtb16	0.000686736	0.663523984
Olr818	1.55461E-15	0.613983739	RGD1561327	3.69539E-06	0.663875684
Antxr1	9.83919E-12	0.614043061	Gbp2	0.000339549	0.664440851
Rab3a	3.46302E-06	0.614648854	Jam2	2.23278E-08	0.665595131

Vtn	5.88183E-07	0.647862094			
LOC690126	9.13613E-06	0.647950471			
Shank3	7.54759E-09	0.648981562			
Npff	6.04251E-08	0.649003909			
Nfe2l3	3.80031E-09	0.649038375			
Heph	5.26846E-10	0.650564429			
Tmem26	8.82188E-05	0.650958886			
Olr1335	0.000374002	0.652372382			
Pik3ip1	3.42647E-06	0.654321137			
Kif13b	4.1337E-05	0.654868098			
PCOLCE2	2.0384E-11	0.655640233			
Kcnb1	5.00948E-08	0.657814179			
Ackr3	1.70448E-05	0.658080986			
Sstr1	8.96371E-06	0.659399794			
Epb41l4a	4.67644E-10	0.660812292			
Dll4	2.85297E-06	0.66086853			
Kcnj14	6.99304E-05	0.661945296			
Bnip3	4.03649E-08	0.662830912			
Pim3	2.39738E-05	0.66390246			
Kiaa0408L	2.92036E-07	0.663986797			
Jam2	1.12233E-09	0.664153993			
Mcam	6.31878E-08	0.664385247			
Hspa8	2.17426E-05	0.664547279			
Herpud1	1.46048E-06	0.664881213			
Ccn3	3.15576E-07	0.66488974			
Arap3	9.04411E-09	0.665157399			
Efnb1	6.99275E-05	0.665240308			
Xkr4	5.32443E-08	0.665473254			
Aqp1	8.43954E-10	0.66555673			

Vegfd	9.19283E-11	0.614997655	Mybphl	3.3765E-10	0.665774724
Rsbn1l	8.61616E-10	0.615669914	LOC102552817	5.63085E-09	0.66619815
LOC102549283	1.14872E-10	0.616212658	Cacnb1	0.000398773	0.666281117
Prxl2c	1.39173E-12	0.617874573	Myoz2	1.29149E-07	0.667690097
Lrrn3	1.0977E-08	0.617905847	Lrrn3	2.22566E-07	0.66824171
LOC102547056	5.68027E-12	0.618008847	Efhd1	3.47344E-10	0.668246461
LOC102549509	6.62117E-07	0.61843427	Tesc	2.60538E-06	0.668638051
Tef	3.62816E-05	0.619012983	Plk1	7.35879E-11	1.494910103
Alox15	1.24546E-07	0.620450483	Rassf4	6.4742E-11	1.49661268
LOC102548807	6.80821E-15	0.620748965	Kifc1	2.49514E-10	1.497459207
Nav2	8.93786E-12	0.621113613	Bpifb5	1.51252E-08	1.497553665
LOC102552104	3.28236E-05	0.621807563	Depdc1	0.000177454	1.498125457
Sept4	4.06334E-12	0.621981648	Bhlhe40	8.59241E-12	1.501607528
Adcy9	2.12468E-10	0.622153975	Ptprn	2.98465E-08	1.501736575
Gpt	2.43663E-10	0.622519262	Letm1	1.01121E-06	1.502402559
LOC102555465	2.74519E-08	0.623445256	Ceacam4	0.000554525	1.50331018
Cpe	2.76121E-07	0.626075313	Tmem54	4.11626E-16	1.504408102
Mfap5	3.63861E-12	0.626593962	Olr1512	1.10964E-05	1.505008268
Sowahb	1.88675E-16	0.627876279	Gpnmb	3.02738E-08	1.507835477
Nat8f5	2.91616E-08	0.630283446	Cyct	0.000570849	1.508406663
LOC102554235	1.00929E-13	0.630357913	Hilpda	1.96903E-11	1.510368185
Fos	0.003833926	0.630840385	Try10	1.42239E-06	1.512367686
LOC108349525	0.000603206	0.631300614	Myo7a	1.23373E-10	1.512537202
Gpr146	3.30444E-12	0.631445566	Ahnak2	1.06438E-10	1.515542463
LOC102549701	9.86121E-08	0.632265643	Cpb2	3.16224E-10	1.51758282
LOC100910187	9.62327E-06	0.632629896	Idi1	6.5302E-09	1.517978722
Ankrd22	5.65896E-12	0.633767653	LOC102554010	7.02284E-08	1.517981809
Cnr1	1.29406E-06	0.633791623	Tmem164	1.77999E-12	1.518226313
LOC100912724	4.97787E-06	0.634172295	RGD1565767	3.17769E-09	1.518526739

Cdkn1c	2.04935E-07	0.666631578			
Cyth3	3.07989E-07	0.666912442			
Crabp2	3.87703E-09	0.66700539			
Pkp1	0.000205386	0.667311856			
Adgrg2	0.000132921	0.667540353			
Trpm5	1.10247E-07	0.667817009			
Prx	9.43414E-06	0.667846717			
Por	1.90374E-05	0.667950904			
Cyba	1.0688E-07	1.495011968			
Fcrlb	5.32656E-05	1.496244501			
Upk3bl1	1.93515E-07	1.496413565			
Paqr5	8.7883E-06	1.497538686			
Fdps	1.71066E-07	1.49843776			
Jak3	6.00797E-08	1.498488716			
Slco4a1	5.09864E-06	1.498501872			
Duoxa1	9.0398E-06	1.498873037			
Slc37a2	2.25254E-05	1.499346483			
RT1-Da	4.00589E-06	1.499471567			
Card9	1.25918E-07	1.500398674			
Fen1	1.88934E-08	1.501112405			
Cdca7l	1.59327E-06	1.501228506			
Ncf2	2.46645E-07	1.504185498			
Tmod4	3.11404E-09	1.506244725			
Scart1	6.99352E-05	1.506965761			
Epdr1	3.09425E-09	1.507364807			
Clec4a	1.21923E-07	1.508593923			
H1f0	3.63661E-07	1.509989239			
Spata46	0.000891657	1.510329411			
Nceh1	1.77676E-10	1.511010168			

Popdc3	8.68425E-12	0.634304011	Ctsh	6.51185E-06	1.518768854
Tac1	0.001185027	0.634371014	Per3	7.32141E-08	1.519442855
Avpr1a	8.72277E-09	0.634720435	LOC103694826	3.31205E-06	1.52192768
Pcdh11x	1.41332E-10	0.635129093	Washc5	8.88564E-05	1.522343546
Cacna1h	5.49875E-12	0.635133343	Matk	2.13571E-06	1.522979404
Ackr4	8.18412E-09	0.635586242	Bmf	1.21813E-09	1.523054702
LOC108348293	4.86076E-08	0.636063909	Slc25a10	1.43703E-08	1.523820072
Rapgef5	5.49875E-12	0.636453205	Tnn	2.94893E-06	1.524429697
Tp53bp2	2.18451E-12	0.637068382	Ldlr	2.82463E-08	1.524810477
Slit1	2.73219E-14	0.637520714	Spdef	5.4568E-13	1.525532526
Fam13a	1.22742E-08	0.637673246	Csrnp1	6.95118E-06	1.525673614
Aldh1a7	3.3685E-08	0.637719712	Card9	2.14611E-08	1.5263394
Itga9	2.15312E-12	0.638429866	Ccdc136	5.77154E-10	1.528559101
Osbpl5	1.67499E-12	0.638576186	Slc7a5	3.61367E-13	1.52968749
Ogn	3.80925E-13	0.638755564	Dgat2	3.51553E-11	1.530483548
Prss23	7.57902E-11	0.639161433	Mc2r	3.84145E-07	1.530904846
LOC103691754	5.66341E-09	0.639231844	Rnaset2	1.57483E-10	1.531351078
Ihh	1.51299E-08	0.639392493	LOC102554845	1.05919E-07	1.531532492
Tns3	4.48655E-15	0.640281088	LOC103691961	2.79071E-12	1.532468394
Spry3	7.48607E-09	0.640822531	Gckr	1.04245E-12	1.532707345
Mab21l4	2.18451E-12	0.641553143	Sfmbt2	2.18638E-07	1.532802594
LOC102548360	1.85968E-07	0.642095787	LOC103692399	6.09021E-05	1.533038252
Dlx2	3.81052E-10	0.642260162	Melk	7.08799E-09	1.533259555
Myrf	1.75627E-12	0.642403529	Kcnn4	2.98436E-10	1.533603557
LOC103690884	1.1853E-07	0.642754085	Il24	8.47219E-08	1.533797876
LOC103693456	5.94024E-11	0.644055799	Scgb1c1	2.79187E-09	1.535125135
Myl4	1.26427E-12	0.645241839	Cyp2c23	3.71026E-09	1.53590316
Akr1c19	8.51951E-08	0.645518194	Gch1	6.16297E-07	1.536462548
LOC100910978	9.49561E-10	0.646500515	Tagln3	1.68078E-08	1.53678023

Cd302	1.10131E-08	1.511667314			
Lonrf3	3.02817E-05	1.512801313			
Nek6	2.21656E-08	1.512966815			
Cd79b	0.000407896	1.51676638			
Slamf6	3.56417E-07	1.517915735			
Serpinb1a	3.19862E-11	1.517918225			
Scimp	1.24761E-05	1.518061791			
Defb52	1.62018E-05	1.518217438			
Malt1	4.82199E-08	1.519544347			
Plk4	9.08195E-06	1.520033143			
LOC24906	4.92905E-06	1.521336237			
Nxpe4	2.19944E-08	1.522291911			
Ppp1r14d	1.92502E-06	1.524019515			
Sit1	6.16502E-06	1.524455139			
Ly6l	8.91867E-09	1.524763083			
LOC102556643	7.26578E-05	1.524847023			
Hsd17b7	5.06206E-07	1.524872215			
Csf2	7.51529E-06	1.526499022			
RGD1564664	6.90066E-05	1.527827581			
Layn	1.15509E-08	1.528227966			
Ccr2	1.11653E-08	1.529171264			
Clec4a3	1.4831E-07	1.53060807			
Ncapg	3.2787E-08	1.530734345			
Ralgapa1	6.56766E-10	1.531084895			
Adm	0.000322298	1.531453978			
Apobr	4.42015E-10	1.531642209			
Ccdc96	7.00977E-09	1.532936367			
Ddit4l2	0.001659131	1.532948306			
Lypd2	6.84328E-10	1.532957591			

Myh11	2.81122E-07	0.646667884	Emilin2	1.75583E-05	1.536842841
Eps8l2	4.08882E-11	0.646683009	Ccl12	7.80277E-05	1.537101764
LOC108350670	7.22556E-07	0.646924269	LOC102553701	1.17468E-06	1.537601876
LOC102548073	3.29587E-10	0.647799416	Slc49a3	1.96166E-08	1.537823665
Galns	1.14656E-10	0.648153411	Vom2r5	1.36292E-08	1.538181279
Stat4	1.45859E-08	0.648373128	Ccl17	3.81272E-08	1.539304568
Camkk1	1.71344E-11	0.649287053	Hmox1	9.92788E-07	1.540012418
LOC498236	7.17206E-14	0.65066027	LOC362863	1.31556E-08	1.542360511
Nap1l3	3.24587E-09	0.650901472	Gna15	1.21237E-08	1.543938138
C1qtnf7	1.72521E-08	0.651890066	Selenow	2.13338E-07	1.545189617
Spag16	9.12228E-09	0.652667775	LOC103692844	1.80858E-09	1.549059287
RGD1564324	5.12597E-13	0.653310985	Vars2	5.37897E-12	1.551899976
Lingo3	0.000102242	0.653494242	Ctse	2.23787E-07	1.552262782
Slc9c2	1.64543E-06	0.654424258	Prl6a1	0.000780636	1.552876352
LOC102552298	1.80051E-07	0.654560159	Fam237a	5.01272E-06	1.554112869
LOC102556302	2.97507E-07	0.654818899	LOC102553345	1.5201E-07	1.554485874
Cdhr3	1.5781E-10	0.654920651	B3gnt7	6.98512E-11	1.554624635
Igfbp5	7.82544E-09	0.654986545	Slc13a2	3.81796E-07	1.556203848
Tmcc2	3.5573E-07	0.655298013	Alas1	4.67597E-10	1.556352229
LOC102553118	2.45129E-10	0.655334042	Pde4c	4.66624E-10	1.55932589
Abcg5	2.832E-06	0.655917627	Hs3st4	9.47451E-07	1.562705671
LOC681177	1.11636E-09	0.656258136	Rrm2	9.81003E-11	1.564336537
Wrap73	9.99912E-08	0.656494159	Mro	2.41815E-08	1.564778412
LOC102547513	2.74939E-06	0.657533478	Wdr91	4.27664E-11	1.564974104
Cdh22	8.11531E-10	0.657973217	LOC103692957	9.08338E-07	1.565036635
LOC103693479	3.95135E-11	0.657974967	Lce1m	5.12836E-09	1.566698568
Rasd2	2.70064E-05	0.658122856	Knstrn	1.07913E-10	1.567859029
LOC103692675	1.34259E-12	0.658186663	Tent5b	4.96169E-05	1.570592567
Lrrc32	8.26816E-07	0.658500202	Slc16a6	2.09826E-07	1.572536703

Clec4a1	6.42041E-07	1.53354528			
C6	0.000117117	1.539741287			
Amdhd2	2.49939E-07	1.540239671			
Litaf	1.52857E-09	1.54142237			
RT1-DMa	9.34318E-09	1.542253588			
Klrc3	7.88985E-07	1.542478576			
Mapkapk3	5.85742E-12	1.543425032			
Bub1	1.13981E-08	1.543593125			
Osbpl8	3.48199E-06	1.54377712			
Parm1	3.28928E-10	1.546366639			
Ccl4	7.30651E-05	1.546620036			
RGD1305807	1.32314E-06	1.546997448			
Cyp51	1.10474E-07	1.548511594			
Lpcat1	3.87703E-09	1.548721086			
Ccl28	1.62461E-05	1.550317437			
Trpv2	5.06206E-07	1.551110621			
Matk	1.62835E-08	1.551162664			
Tmem132c	4.28172E-09	1.554255066			
Gas2l3	1.26165E-08	1.556138097			
Selp	8.43703E-07	1.556576554			
Bcap29	1.14885E-10	1.556613507			
Ncf4	8.12684E-08	1.556734035			
Mcm3	4.2592E-07	1.557202355			
Gabrr2	1.10572E-05	1.559504669			
Siglech	5.45049E-05	1.561270325			
LOC103690190	7.36775E-08	1.564048475			
Prc1	1.95845E-09	1.56513195			
Icos	4.82886E-11	1.567269432			
Ccr5	1.06571E-07	1.568235525			

Smpx	9.98844E-08	0.658836553	LOC103691740	8.39129E-09	1.575192548
Sfrp2	2.91339E-09	0.659053943	Tchh	1.39617E-05	1.576078596
LOC102552887	1.85715E-14	0.659380948	Ly6i	1.05354E-05	1.576878636
Mylk	2.78135E-10	0.659684593	Slamf8	1.06438E-10	1.577738408
Vldlr	5.5887E-10	0.659780772	Arg1	1.84708E-10	1.577779934
LOC103692511	2.65457E-08	0.659880215	Muc20	9.99408E-09	1.577998031
Rhobtb1	2.85293E-13	0.660589746	LOC108349815	3.14377E-06	1.578401729
PCOLCE2	1.16409E-09	0.660775377	Cxcl6	2.84523E-08	1.58003018
Ddit4	0.000434638	0.660982974	Lyc2	1.6867E-08	1.580739279
Neu3	2.46692E-12	0.661303265	Dhcr7	3.97224E-09	1.582363883
Vom2r2	2.97995E-08	0.662000415	Prkar1b	1.10482E-07	1.582862324
Fam151a	1.74897E-11	0.662039321	LOC103690379	1.27744E-08	1.583157096
Reck	1.0268E-14	0.662251396	C4a	1.23098E-06	1.584646469
Tnik	4.0691E-08	0.662501582	Prmt8	1.4218E-07	1.585396146
Olr1748	1.97123E-11	0.662885379	Gal3st1	1.30179E-11	1.588756703
LOC103693970	3.72176E-08	0.663100727	Vom2r4	9.36947E-05	1.590065374
LOC100911572	2.46692E-12	0.663204644	Spc25	7.85202E-12	1.590486974
Sned1	4.62711E-09	0.663525785	Tnfaip2	1.6104E-12	1.592459529
Dcdc5	3.4132E-09	0.663623128	Bpifa5	2.28661E-07	1.594414046
Nmb	0.000382847	0.663734437	Akt1	5.28612E-09	1.595083558
Depp1	0.003266329	0.663871382	Fbp2	3.87945E-10	1.595482905
Syde2	8.74928E-09	0.664159864	Atp8a1	1.80697E-08	1.597173416
Stat6	4.01575E-14	0.664782731	Cks2	6.43151E-11	1.597515107
Ablim3	1.86465E-12	0.66487265	Foxs1	5.0175E-08	1.600430298
LOC103691437	2.8656E-08	0.664984039	Lmln2	5.08505E-05	1.601805655
C1qtnf2	7.03361E-10	0.665696228	Pip5k1b	9.35364E-08	1.601974019
RGD1563738	0.000434457	0.665723373	Sord	3.15475E-10	1.603498598
LOC102554207	3.73962E-05	0.66579388	LOC100363535	3.68757E-07	1.603783747
Fosb	0.004650431	0.665962314	Upk3bl1	4.50195E-11	1.603911847

Cdh17	7.39521E-08	1.570770361			
Folr1	5.92791E-07	1.571316092			
Acbd7	0.00016549	1.571945568			
Troap	1.09099E-05	1.574330593			
Slc23a1	2.61411E-08	1.574759518			
Gpr137b	8.33771E-08	1.575393598			
Itgb8	1.88934E-08	1.578161938			
Evi2a	4.19117E-07	1.579296706			
Depdc1	4.50285E-07	1.579718931			
Retnlb	1.84131E-07	1.581153784			
Cdc20	6.56766E-10	1.58125405			
Cdc45	2.72187E-08	1.582597726			
Sct	7.24841E-06	1.582763257			
Il7r	1.01806E-09	1.582905442			
Pkmyt1	0.003140362	1.58506532			
Tjp3	5.9946E-10	1.590970689			
Dlgap5	6.84328E-10	1.591097275			
Cd3g	6.16203E-06	1.592139229			
Pbk	3.04506E-08	1.592635932			
Pappa1	7.98187E-07	1.592823024			
Bri3bp	2.94938E-09	1.59387087			
Aqp3	0.004849511	1.594003847			
Pf4	7.60626E-08	1.594166587			
Gfer	2.45114E-07	1.594350212			
Pip5k1b	9.47045E-10	1.594527119			
Capg	1.03403E-07	1.594578762			
Cyp7b1	2.36258E-06	1.594894532			
Arg1	7.22519E-06	1.596585096			
Rnaset2	2.30785E-10	1.597046262			

LOC102552849	1.95293E-06	0.666005428	Klk13	5.60928E-12	1.60546233
Clec19a	1.40638E-08	0.666188208	Ect2	3.86943E-10	1.608018515
Mcpt9	1.6894E-05	0.666464207	Iapp	4.69813E-09	1.608842264
Mfap4	3.88822E-07	0.666551238	Tef	4.05357E-05	1.610696444
Ndn	2.31478E-10	0.666631997	Card14	1.1535E-08	1.611011201
Efemp1	1.48337E-11	0.666873825	Pdzk1ip1	1.77144E-07	1.611628079
Fxyd1	5.74074E-08	0.667546375	Ppic	9.49902E-13	1.611634591
LOC100912221	1.66126E-09	0.668182977	Nxpe4	2.39291E-09	1.614216336
Cyp2f4	2.03408E-08	0.668427803	Hist1h1b	4.8696E-08	1.614841893
LOC103691422	2.20597E-11	0.668546156	LOC102549526	2.17288E-07	1.616889741
Cks1b	3.61267E-10	1.495022894	Chst1	4.80757E-10	1.617419411
Tmem171	2.35449E-10	1.497807817	RGD1305807	1.46456E-11	1.617598018
Slc6a12	5.24282E-09	1.498602374	Nr4a3	1.10382E-09	1.618052085
Bub1b	1.02977E-09	1.498762996	LOC102548928	5.19158E-10	1.619159124
Tpp2	3.61143E-05	1.500384532	Cftr	3.1148E-09	1.619442426
Olr1750	6.39723E-07	1.501246294	Dpp7	1.20305E-08	1.619791249
Tubb6	1.76256E-10	1.502007793	Mt2A	1.40511E-06	1.619895678
Gpr37l1	3.88037E-10	1.502109058	Olah	9.41483E-08	1.620656438
Atp8a1	1.4612E-07	1.50418557	Dazl	2.90501E-07	1.622968306
Ska1	1.61644E-07	1.505302885	Vom2r1	1.37007E-06	1.623363516
Mmp14	2.34399E-08	1.50536749	Gpr183	6.07817E-09	1.625168603
Endod1	4.12779E-12	1.506753917	Cdkn3	3.51553E-11	1.628706622
Kcnj13	1.43499E-09	1.507575111	LOC103693315	0.001889037	1.630223098
LOC100912070	0.00375095	1.509507424	Rad51	6.43151E-11	1.630669712
Olr1	5.01257E-06	1.509891334	Mcc	1.4201E-05	1.630744669
Mis18a	7.16618E-07	1.509930533	Hpx	1.75339E-07	1.63172186
Tnfrsf12a	1.05028E-06	1.511992219	Sema4a	3.03888E-10	1.632258111
Bpifb2	2.70991E-09	1.512098492	Baalc	1.53644E-06	1.632340624
Adra2a	2.92387E-06	1.51353926	LOC102548331	2.69491E-10	1.634069819

Pak1ip1	6.56766E-10	1.597191778			
Ccno	6.67156E-12	1.598443433			
Slc2a1	4.33676E-09	1.599069933			
Mylk2	6.64227E-08	1.599925226			
Mybl2	5.41759E-10	1.599978285			
Rmdn2	1.10925E-10	1.601437698			
Cd68	1.17251E-06	1.602163356			
Mvd	1.57603E-08	1.602486133			
Arhgap11a	1.08244E-09	1.602593346			
Tmem45b	1.86652E-06	1.603038434			
Gkn3	3.78766E-06	1.603843991			
Dhcr7	1.02709E-06	1.605446443			
Bcl3	2.48707E-07	1.606912063			
Ccr6	1.70151E-06	1.610501228			
Hist1h2ail1	5.12151E-08	1.611412913			
Stambp	1.65069E-05	1.611932108			
Akr1b8	4.71213E-11	1.612010839			
Prss22	2.4024E-08	1.612096343			
Cxcl11	0.000204347	1.6145955			
Hpdl	5.37916E-10	1.615995163			
Sqle	8.00666E-07	1.618638538			
Ifi30	8.22797E-08	1.619757374			
Lst1	2.37734E-07	1.620524231			
Ch25h	0.005433932	1.623372549			
Shisa8	2.91497E-08	1.628233766			
Diaph3	1.05383E-10	1.629100361			
Cfb	4.33056E-13	1.62942795			
Myo5a	2.92505E-07	1.63021254			
Dsn1	1.13183E-05	1.632550492			

Muc20	4.20378E-08	1.513662863	Retnlb	3.1985E-08	1.634325043
Isg15	6.15436E-08	1.514812959	Fcrlb	1.28124E-06	1.638754779
Hk3	1.92307E-09	1.51560433	Meis3	1.58456E-08	1.643969911
Prss22	2.82616E-09	1.516175893	Tmem173	1.37992E-10	1.644377517
LOC102554948	1.28179E-08	1.519498721	Zbtb48	4.12799E-07	1.646093254
Cldn10	6.91245E-09	1.519819187	Tspan11	1.60659E-10	1.652576155
Fam187b	7.63478E-12	1.520186699	LOC108349525	0.000242514	1.653408488
Slc11a2	7.50676E-12	1.52275184	Hmgcs1	1.09742E-13	1.654548813
St3gal4	4.7049E-12	1.523157289	LOC103694928	1.66902E-10	1.655137714
Hist1h1d	1.69346E-06	1.523596057	LOC102555378	3.11711E-08	1.660952532
Gpr84	1.00062E-10	1.523716976	Slfn13	3.85337E-07	1.664849926
Rab7b	3.73586E-09	1.529445607	Il1r2	3.63516E-07	1.667267537
Cip2a	4.22333E-08	1.530015518	Qsox1	4.04805E-09	1.667939183
Icos	3.30214E-13	1.530029681	Pcnx2	6.68389E-16	1.668291502
Cenpt	1.9423E-13	1.53191353	Areg	5.68256E-05	1.675396296
Fen1	4.04631E-16	1.53291052	Vwa7	3.59059E-06	1.678635688
Ccl22	1.13518E-11	1.533532884	Cenpw	3.36387E-11	1.678797419
Rhbdl2	4.97541E-11	1.535179503	Rab7b	1.43057E-10	1.682160848
Dennd2d	4.23203E-11	1.535401595	Siglec1	7.46144E-07	1.685529777
LOC103691845	9.81619E-10	1.536784469	Tpcn2	0.000177105	1.68984894
Npc2	4.44761E-07	1.538759157	Muc1	1.53322E-07	1.692862591
Tmem45b	4.64455E-06	1.538787442	Olr86	1.25757E-05	1.694232043
LOC102554972	0.000176506	1.539237901	RGD1565617	0.000352475	1.694618926
Adm	5.27519E-06	1.539773061	Hk3	3.66256E-11	1.701049851
Krt15	0.000496746	1.542086566	Sfta2	7.93063E-12	1.707705469
Susd3	3.56755E-10	1.542794561	Kcnip4	3.17885E-11	1.707790252
LOC102546495	1.58143E-07	1.542869958	Lpcat1	4.67118E-06	1.71015127
Defb5	9.3424E-05	1.542958607	Obsl1	9.70714E-09	1.71185605
Shcbp1	3.69794E-07	1.544986649	Tectb	2.09234E-11	1.717614349

Fxyd3	6.05229E-13	1.63290755			
Gabrp	5.4156E-09	1.636019058			
Qsox1	3.18256E-10	1.637862337			
S100a14	2.43833E-11	1.639347623			
C4bpb	5.24335E-06	1.640588039			
Tubb2b	4.0153E-11	1.641284041			
Cenpf	1.15026E-12	1.642897366			
Tf	1.17887E-08	1.646810274			
Sema4a	1.09687E-07	1.647091697			
Klk13	8.12929E-09	1.647290362			
Acsl4	1.14885E-10	1.647642314			
Ms4a6a	1.21219E-07	1.649590057			
Ebi3	1.18237E-09	1.65178514			
Ska1	7.31818E-09	1.652432141			
Cdca2	0.000191973	1.652433767			
Slc6a12	2.43282E-08	1.654317222			
P2ry6	3.61205E-09	1.658541057			
Cotl1	6.36091E-10	1.659834934			
Mad2l1	1.84388E-10	1.661519562			
Cep72	1.47208E-06	1.668123311			
LOC684828	0.00032516	1.668930428			
Racgap1	7.71812E-11	1.672631122			
Slc6a14	1.44979E-10	1.673472661			
Tnfrsf17	0.00010114	1.673750756			
Mbl1	2.1766E-10	1.675512466			
Hist1h1d	3.1261E-06	1.675599407			
Hist1h2ai	4.62928E-05	1.676190101			
Prmt8	1.59E-05	1.676322378			
Dctpp1	4.07993E-09	1.678085154			

Dmkn	0.001666906	1.54512551	Gmds	1.36398E-06	1.720931392
Ifit3	4.4418E-11	1.545952006	Rmdn2	5.66E-10	1.729917361
Gins1	1.08636E-11	1.546317874	Fcgr2b	7.37839E-08	1.731289615
Plk4	1.24346E-13	1.546369595	Igll1	9.3022E-07	1.735766773
Dlgap5	2.45325E-12	1.546943435	Notch3	7.40392E-09	1.736402469
Tfap2e	1.75216E-07	1.547739187	Ccl3	1.37892E-10	1.739552397
Asf1b	1.30324E-13	1.554068503	Bhlha15	5.14389E-13	1.740104658
Ksr2	0.000355291	1.558053668	Chdh	3.66205E-12	1.740467407
Tnfrsf9	2.24564E-08	1.558097472	Rarres1	8.69263E-08	1.740729781
Kntc1	5.12597E-13	1.558782246	Ereg	7.71161E-06	1.740855332
Mbl1	6.27695E-18	1.55887717	Car5a	1.07234E-13	1.741600074
Dsn1	3.22976E-09	1.559819796	LOC102555525	1.11101E-09	1.74440897
Layn	9.47914E-14	1.559841649	Otulinl	5.84091E-10	1.74525402
Pcyox1l	3.56535E-09	1.559990307	Tac4	1.81707E-08	1.749506038
Cpt1a	7.15493E-10	1.560223124	LOC102555435	0.006183163	1.749599164
Scgb3a2	1.98849E-10	1.562815808	Emid1	0.000738915	1.751649843
Mcm5	4.33937E-12	1.563848343	P2ry2	1.10267E-11	1.751973024
Zbtb48	1.85853E-06	1.564439461	Gjb6	2.28385E-11	1.755030587
Tnf	5.91555E-10	1.568164478	Edil3	6.02388E-10	1.755977234
Pdss1	1.44429E-09	1.568465722	Col16a1	5.25998E-11	1.760328253
Tnfsf9	2.66702E-08	1.568619714	LOC103691630	5.82852E-11	1.76042935
LOC102547753	1.7986E-05	1.568731248	Sct	1.28702E-10	1.760583225
Cenpu	3.50017E-11	1.569091677	Rhox2	7.61335E-07	1.767532265
RGD1564664	1.44648E-07	1.569224151	Serpine2	6.10342E-09	1.771422308
Serpina10	9.45899E-14	1.569723071	LOC102547753	6.59712E-07	1.772319753
Asb18	2.10226E-06	1.572299766	Nmb	6.91211E-06	1.772621022
LOC102555648	9.96576E-10	1.573540735	Vsig2	3.4546E-08	1.774056093
Rorc	4.45897E-15	1.574601827	Mab21l3	3.35736E-09	1.775501113
LOC108351280	3.96481E-11	1.575594483	S100a9	3.33961E-06	1.778829656

F2	1.96588E-08	1.679434837			
Dmkn	9.52698E-06	1.682645705			
St3gal4	1.26881E-08	1.684233013			
Rad51	3.4533E-08	1.686956172			
Casp4	3.82409E-11	1.687155179			
Rassf4	1.53416E-07	1.687324691			
Nfil3	1.44608E-07	1.691506455			
Cldn10	5.98198E-13	1.691570151			
Dennd2d	5.44381E-12	1.691666954			
Hpx	7.70242E-09	1.693009846			
Baalc	1.12233E-09	1.694367692			
Angptl4	0.000308354	1.695912813			
LOC684797	2.48968E-06	1.701911931			
Il1rn	1.43806E-08	1.701960574			
Serpina11	2.04335E-05	1.702894164			
Rab7b	3.36023E-07	1.704272473			
Gja1	1.04139E-10	1.712820511			
Crtac1	7.36528E-08	1.714469892			
Muc1	3.67175E-12	1.715979896			
Tlx3	0.007285497	1.719709801			
Dusp2	9.11248E-08	1.726255935			
Mis18a	2.30785E-10	1.729959764			
Gpr183	1.85507E-08	1.733563348			
LOC683313	4.00644E-07	1.736646082			
Fcgr2b	2.72187E-08	1.738982853			
Zbtb48	6.66275E-10	1.741348469			
Kntc1	1.21799E-09	1.743889296			
Entpd3	4.72643E-11	1.744343821			
Cenpu	3.56969E-11	1.749960222			

Zfand2a	1.12523E-13	1.576090032	Snhg11	6.4742E-11	1.779433465
Spink2	2.34721E-07	1.57796322	LOC100912312	8.69537E-14	1.781025117
LOC100363535	5.20809E-07	1.581234788	Lhfpl2	2.93117E-11	1.78197102
Tmem56	2.98278E-10	1.581839987	Bhlhe41	2.61302E-13	1.784877251
LOC102555226	7.19752E-12	1.58269894	S100a14	1.29003E-10	1.786305609
LOC100912312	2.57989E-12	1.583109015	Serpine1	1.98761E-08	1.787448672
Plppr5	1.02251E-05	1.583946553	RGD1565367	3.9448E-10	1.787706966
LOC103691483	7.82659E-10	1.584352783	Adora2b	1.01726E-09	1.789601381
RT1-M2	2.48182E-08	1.584494004	F3	9.22205E-07	1.791889635
Foxq1	2.54766E-09	1.584669011	LOC100360128	7.93063E-12	1.792081009
Irf7	7.35538E-09	1.584835223	Brs3	7.06039E-12	1.797977955
Prr18	6.22382E-14	1.585132583	Cdca3	1.76E-12	1.800802115
Hist1h1a	4.81575E-07	1.585950431	LOC103690357	1.62839E-08	1.801957094
Ly6c	1.49902E-05	1.587832009	Cfb	8.28479E-08	1.811715701
MGC105649	2.77756E-06	1.588577174	Gdf15	1.0094E-08	1.815090451
S100a8	6.47813E-07	1.589679911	LOC102556856	1.64468E-08	1.817732007
Elf3	2.53124E-12	1.589807013	Defb43	0.00041706	1.819186012
Espl1	1.10819E-10	1.59116061	Shcbp1	2.25919E-09	1.821657169
Qsox1	1.61958E-08	1.594245017	Tmem86a	3.39811E-11	1.822464071
Cry1	2.73254E-13	1.594330162	Slamf9	7.86733E-09	1.832060467
Tmprss4	2.43935E-11	1.595066058	Sapcd2	5.33195E-12	1.834596507
Rundc3a	6.28389E-12	1.597200799	Fetub	7.15425E-10	1.836684141
Mybph	5.73558E-13	1.59753819	Myom3	7.75309E-09	1.837012205
Fam111a	3.72328E-12	1.599510502	Spetex-2H	1.31018E-12	1.840488863
Tlr2	4.38428E-18	1.601750794	Sel1l3	4.65913E-10	1.840892462
Faim2	1.57328E-08	1.602436383	Tnfsf9	1.79412E-10	1.841724149
LOC100909946	2.86532E-14	1.606999929	Gadd45g	6.96483E-08	1.851676575
Brms1l	3.47224E-06	1.607022751	Acot1	8.58606E-06	1.852848267
Enpp2	6.13229E-11	1.608817415	Bcl2l14	2.99443E-12	1.856614234

Bhlha15	4.89976E-07	1.751010356			
Itgam	2.49891E-07	1.751329623			
Tpx2	3.60918E-07	1.75266506			
Abhd11-as1	5.37916E-10	1.755425342			
Notch3	1.13877E-08	1.75857154			
Idi1	1.75486E-07	1.76480404			
Hmgcs1	1.30376E-06	1.764976888			
Igll1	0.006217357	1.770628567			
Hk3	1.50616E-05	1.778946034			
Hist1h1a	1.3351E-09	1.784974019			
Tac4	2.60178E-10	1.789379952			
Birc3	3.29852E-10	1.789481673			
P2ry2	2.2057E-09	1.792746329			
Dscc1	2.16111E-09	1.793058861			
Bhlhe41	1.76276E-10	1.797304153			
Gadd45g	3.99502E-05	1.797375212			
Tmprss4	6.11483E-10	1.797426787			
Sele	7.01561E-06	1.8001556			
Tnfrsf11a	8.13631E-10	1.802330841			
Muc20	4.48181E-10	1.804599308			
Rnd1	8.66567E-10	1.807463342			
Kif15	9.59186E-11	1.808038561			
Duox1	9.22197E-08	1.809757937			
Phgdh	3.18278E-09	1.811559069			
Bard1	7.07375E-10	1.813429029			
Tspan11	7.07375E-10	1.814439947			
LOC689757	0.003269807	1.820834042			
Nipsnap3b	2.09107E-11	1.821987148			
Tekt2	1.72827E-10	1.828782147			

Batf	6.12006E-10	1.610516494	Grin2c	1.99133E-14	1.858009655
Cenph	4.64314E-15	1.612464024	Ank3	2.51075E-12	1.860643169
Bcat1	7.58865E-09	1.613520308	LOC103692085	1.06782E-10	1.863299747
Esr1	7.50197E-06	1.614659954	Entpd3	3.75263E-10	1.868833928
Rnaset2	1.313E-11	1.615547659	Cited2	1.67748E-10	1.870520313
Vsig8	1.2175E-10	1.616379771	Mreg	2.25996E-12	1.872096131
C1qc	3.48445E-08	1.61911029	LOC100364194	1.69991E-09	1.890481626
Hilpda	6.11871E-13	1.620111914	Vsig4	9.80525E-07	1.897361355
Unc5cl	2.18698E-05	1.620148971	Fstl4	1.84557E-11	1.901411456
Hpx	1.90349E-07	1.621338053	Try5	2.01379E-08	1.903899669
Trappc3l	4.92917E-08	1.621446664	Abca6	1.87473E-10	1.905562511
LOC102555869	1.16499E-07	1.621742628	Per2	4.99728E-07	1.906690252
Kcnn4	2.20597E-11	1.625738701	Ovch2	0.000963827	1.91929123
S100a14	2.18782E-09	1.625815426	Slc7a11	6.03909E-11	1.922021932
Usp18	1.99045E-11	1.628011074	Nxf7	5.99274E-11	1.922720143
Otulinl	4.36593E-09	1.631894687	Tnfrsf17	1.39367E-07	1.927281858
Kif20b	7.7468E-11	1.635322363	Mmp12	3.57554E-05	1.928211799
LOC102550017	1.59013E-14	1.635602942	Daam2	4.03652E-11	1.929826552
Bhlha15	2.15312E-12	1.636739424	Zfp507	2.85946E-06	1.936475925
LOC103692235	0.000248164	1.641620716	Chi3l1	5.73961E-07	1.937427752
Muc4	2.53728E-13	1.643349382	Cd5l	5.13733E-09	1.941337318
Itgam	5.55252E-11	1.646978969	Col9a2	7.54146E-09	1.94312512
Rasgef1c	2.54929E-15	1.651651133	Lcn2	5.41277E-05	1.971267669
Melk	3.45936E-10	1.651722731	Fhod3	5.43992E-10	1.974557572
Fabp4	2.69737E-06	1.652823169	Slc7a7	2.64689E-08	1.981698687
Cfb	9.74921E-07	1.653134507	Nr1d2	7.75735E-08	1.987918212
Ceacam19	3.36934E-11	1.654292984	C4bpb	9.01273E-12	1.989257623
Cxcl10	0.000725274	1.654332612	Ammecr1	1.22489E-08	2.000398062
Ube2l6	1.67301E-09	1.654753177	Ccl1	5.34272E-08	2.000860683

Pdzk1ip1	4.94315E-11	1.836515279			
LOC100911796	1.30729E-09	1.836609632			
Mmp14	5.3603E-13	1.843589468			
Fam3b	1.2784E-06	1.843896236			
Asf1b	4.32706E-09	1.845958234			
Cd14	6.67156E-12	1.851080965			
Atp8a1	1.34611E-08	1.856231305			
Ccl3	2.12779E-06	1.858239363			
Gins1	2.78274E-08	1.85984043			
Brms1l	2.72593E-11	1.86455388			
Mcm5	3.87703E-09	1.86583276			
Cxcl10	0.005234745	1.884211889			
Slc16a3	1.12233E-09	1.891960171			
Kif20b	6.67156E-12	1.896923381			
Ceacam4	1.82549E-08	1.897940621			
Gpr37l1	1.31632E-11	1.901555126			
Bub1b	1.23691E-09	1.906402309			
Susd3	3.8103E-11	1.909160565			
LOC102549173	9.07381E-11	1.914681108			
Arntl	4.97954E-06	1.924245883			
Slamf8	8.72661E-10	1.941558066			
Clca1	0.00347496	1.942108901			
Rasgef1c	7.46197E-09	1.944962927			
Retnlg	1.46972E-06	1.955949636			
Fetub	4.38832E-14	1.963496674			
Ctsk	1.23802E-06	1.969301707			
Batf	3.49287E-10	1.974989625			
Cthrc1	7.86071E-11	1.975342561			
Gpr34	6.76289E-07	1.975547473			

Acot1	9.61114E-05	1.662304446	Prss2	2.94339E-07	2.001001767
Ebi3	6.47895E-16	1.664341869	Lilrb4	1.6104E-12	2.018198101
Dio3	6.89189E-08	1.664517382	Pla2g4e	2.51309E-09	2.025045328
LOC108348907	1.95293E-06	1.666484132	LOC100363510	3.04254E-05	2.029560509
Gk	5.10193E-10	1.666485984	LOC689770	3.27995E-12	2.038557452
Fabp6	1.81997E-08	1.667230466	LOC690813	7.6093E-09	2.03929923
Retn	9.82791E-08	1.668425364	LOC102552667	0.000127026	2.049751908
LOC102548847	5.39012E-11	1.66905295	Ankrd33	0.000475673	2.051337465
Itih4	6.40706E-11	1.672031146	Krt80	0.002616618	2.051385822
Cxadrl1	8.76244E-08	1.672970828	Ocm2	0.000497793	2.067928577
Bard1	6.29124E-17	1.674583397	Rhox5	8.29218E-07	2.070689905
Hmox1	5.39269E-08	1.676362368	Itgam	1.09742E-13	2.072754113
Pdzk1ip1	4.31886E-08	1.676881972	LOC103693724	8.50139E-05	2.082445372
LOC103692795	1.69565E-08	1.677499619	LOC689757	1.01055E-12	2.10775202
RGD1565410	2.41214E-07	1.678449591	Cxadrl1	2.00562E-10	2.108431812
Gadd45g	8.48015E-07	1.683309	Mylk2	3.51553E-11	2.109849942
Cdkn3	6.20508E-12	1.686576463	C4bpa	3.96192E-09	2.124861061
Sct	3.6036E-10	1.689103469	Atp6v0d2	4.95747E-12	2.137836818
Mylk2	1.1546E-08	1.692992972	Cdh17	2.69162E-13	2.141412735
LOC102551847	4.61712E-05	1.69428316	Brms1l	2.10949E-09	2.145437655
Bmf	1.5958E-11	1.695645895	Galnt5	1.36176E-09	2.147998437
Clmn	9.82943E-11	1.695836932	LOC102551304	5.20103E-07	2.155985548
C4bpb	6.65152E-10	1.696068372	C3	2.0907E-09	2.172615404
C3	8.98242E-07	1.696919772	LOC103691483	1.09742E-13	2.174842787
Ccna2	2.92195E-15	1.699232764	Ly49s5	4.96647E-06	2.181746042
LOC102555378	1.28487E-08	1.700784882	Duoxa1	4.87998E-11	2.184420871
Pvt1	4.72507E-09	1.70426691	Ccdc116	1.5637E-07	2.203331006
Clec10a	2.33538E-09	1.71119722	Hp	9.91394E-11	2.215762067
Siglec1	4.17972E-07	1.713183499	Itih1	2.17545E-07	2.226085293

Slc7a11	4.22703E-12	1.982196427			
Chaf1b	2.47547E-08	1.994397264			
Lilrb4	2.43833E-11	1.998839117			
Ccl22	1.84388E-10	1.999302281			
Melk	5.90967E-08	1.999964587			
Fgg	3.53355E-12	2.003768292			
Tnfaip2	4.31157E-10	2.006865913			
Ubd	3.2056E-06	2.011991117			
Degs2	3.80515E-10	2.015325596			
Tmem56	4.71665E-13	2.015466337			
Fabp5	6.42586E-09	2.030798968			
Car8	2.41682E-13	2.03397033			
Noxa1	1.15026E-12	2.03543809			
B3gnt7	9.34105E-13	2.035889356			
Espl1	2.43833E-11	2.03639525			
Slc7a7	6.40651E-11	2.046533701			
Tk1	4.87333E-11	2.052376527			
Nuf2	1.85563E-10	2.057863559			
Defb3	5.24624E-09	2.070648617			
Gch1	4.22703E-12	2.076298639			
Gk	1.16607E-14	2.079167048			
Ndc80	3.20343E-10	2.090832979			
Il1r2	5.61754E-07	2.097967445			
Aspm	4.71213E-11	2.099250419			
Tlr2	3.20343E-10	2.102055843			
Bpifa5	8.6071E-12	2.110759302			
Knl1	4.71665E-13	2.111649194			
Ptx3	1.75331E-13	2.114794928			
Ccl9	8.64346E-08	2.114935523			

LOC102547081	2.31765E-08	1.71407689	Rgs1	1.11867E-11	2.243043493
Sel1l3	2.94477E-09	1.718706669	Tmem45b	1.98219E-10	2.254203367
Ctsk	4.29665E-11	1.720456398	Ccl2	7.38768E-12	2.279788069
Mt1	1.30473E-11	1.721144288	Esr1	1.54429E-09	2.293381908
Defb3	3.05828E-10	1.721342566	Dio3	1.39983E-11	2.339733839
C9	5.65896E-12	1.722400877	Fcrl2	1.26134E-10	2.36075064
Tmem173	1.91541E-11	1.723825508	Ccl7	7.24393E-10	2.410543188
LOC685762	2.43935E-11	1.724706653	Ctsk	1.47952E-14	2.421399914
RGD1562462	8.60888E-08	1.724765184	Rasd2	2.28986E-10	2.437614981
Serpine1	4.77059E-08	1.726313882	Cxcl2	6.5302E-09	2.460512213
Etnk2	5.13458E-09	1.726752317	RGD1565655	9.1121E-09	2.486734865
RGD1565655	1.64029E-05	1.735875427	Prss30	3.85206E-13	2.519036035
LOC102548624	2.14836E-11	1.737729308	LOC102555481	5.74229E-12	2.525663197
Ly6al	1.58238E-06	1.740448547	Duox1	7.29897E-11	2.553763436
Kifc1	4.73045E-13	1.74358814	Cd177	1.55382E-07	2.561778586
Kif15	3.85564E-09	1.744735063	Lce1f	1.16978E-16	2.620998286
Bub1	5.29735E-14	1.745206496	Defb5	4.82332E-10	2.656928021
Notch3	5.18113E-09	1.746393855	Gsg1	2.22623E-08	2.681468082
LOC100910424	4.04631E-16	1.751705621	Ptx3	7.47963E-13	2.697272797
Tspan11	1.48436E-11	1.754589905	Try4	5.22342E-10	2.699767337
Fcrl2	1.15689E-07	1.763310607	Kng2	4.98385E-11	2.738270467
Il1rn	9.84072E-09	1.764536161	Tbc1d1	1.43698E-06	2.835945962
B3gnt7	3.95678E-13	1.76574834	Lce1l	1.37276E-14	2.95237374
Il11	1.0907E-10	1.766875449	Nr1d1	1.22489E-08	3.191437382
Mapk10	2.33868E-08	1.769722446	Bpifb1	1.44083E-08	3.209105171
Ebf2	2.66361E-08	1.770825907	LOC102550137	5.20869E-14	3.403715589
Cx3cl1	4.85836E-12	1.772611158	Cfi	1.20144E-10	3.52404746
Chst1	1.52771E-11	1.774295901	LOC102552128	1.35352E-14	3.56512388
Rarres1	4.28722E-08	1.7752719	Scn10a	7.06039E-12	3.69956321

Elf3	7.5222E-11	2.127984809			
Sapcd2	3.44469E-12	2.13620184			
Kifc1	1.63107E-11	2.138298792			
Srxn1	3.60217E-12	2.138310489			
Tuba8	8.88924E-08	2.139885744			
Defb4	4.94315E-11	2.146908606			
Bcat1	6.72582E-10	2.146953276			
Ckap2	2.27105E-13	2.152798043			
Top2a	6.67156E-12	2.154351317			
Prkar1b	9.34105E-13	2.162331241			
Ly6c	3.4123E-11	2.193526032			
Sel1l3	5.15419E-14	2.202059322			
Atp6v0d2	8.52409E-08	2.208619469			
Fcrl2	3.87703E-09	2.211574681			
Col9a2	6.92271E-13	2.238948934			
Vsig8	7.02901E-11	2.242123804			
Calcb	4.37786E-09	2.2436691			
Mt1	4.71665E-13	2.243764179			
Enpp2	5.00531E-14	2.244641392			
Kif11	5.08348E-12	2.246454144			
Scn10a	4.25565E-10	2.247815442			
Ube2t	4.56994E-11	2.255913762			
Ccl20	4.53468E-09	2.259230825			
Ankk1	1.36152E-13	2.261310261			
Cenpt	2.60178E-10	2.267870406			
Mki67	5.9398E-10	2.269568813			
Enpp3	2.30785E-10	2.270770405			
C9	7.93204E-12	2.274753213			
Ccne1	1.70533E-10	2.275901338			

Vsig2	2.7251E-08	1.779917825	LOC102551003	1.61346E-09	3.725429955
Gdf3	2.76934E-09	1.780106148	Mmp7	4.8179E-14	3.797973675
Retnlb	1.60307E-09	1.786546732	Slc26a4	4.77228E-09	4.175888096
LOC300024	1.81997E-08	1.787363832	Aqp3	5.73199E-09	4.431130911
Fcgr2b	2.49097E-08	1.78847308	Kng1	3.21471E-11	4.624970816
Srxn1	2.15606E-15	1.794071904	Chia	7.49184E-12	4.631143422
Cxcl11	6.12851E-06	1.796691736	RGD1309110	4.14569E-12	4.847851111
Fcrlb	8.1021E-08	1.797838961	Dbp	2.83569E-09	5.884288612
Rnd1	7.72263E-10	1.798259859	Lpo	5.39482E-12	6.399489142
Fetub	1.03432E-09	1.798718572	LOC103691629	1.23565E-12	8.021306527
Rad51	1.41757E-12	1.799335153	LOC100911498	2.54304E-05	8.048179529
Ckap2	1.11852E-13	1.800729353	Spp1	2.72325E-10	8.859286533
Cxcl1	4.35628E-06	1.801058626	Saal1	5.26287E-14	11.01610402
Slc7a7	2.54117E-07	1.802416207	Orm1	1.47714E-14	12.03940584
Serpina3n	4.90338E-08	1.805504091	Retnla	1.89793E-11	12.14626549
Atp6v0d2	2.74719E-10	1.806678603	LOC103691626	6.20417E-14	12.55432278
Lce1l	2.78772E-10	1.809072033	LOC102549724	2.97937E-14	17.69983356
LOC102554819	2.77105E-11	1.81369182			
Kif18b	6.91912E-12	1.815052989			
Aspm	4.26497E-16	1.816274852			
Ccnb2	3.07997E-13	1.817206822			
Shisa8	1.10118E-11	1.8309857			
Top2a	1.11852E-13	1.832656271			
Ifi27l2b	6.46566E-07	1.832765177			
Noxo1	1.43146E-05	1.846301528			
Ptx3	2.31911E-09	1.846699914			
Cep55	7.0948E-06	1.850169924			
Armc12	1.78081E-11	1.870459683			
Nuf2	4.28701E-14	1.871614379			

Ttk	3.95939E-10	2.290304649			
Ccnb2	7.44823E-10	2.314009587			
Kif18b	1.14499E-12	2.314556215			
Slamf9	6.40651E-11	2.318916155			
RGD1565410	5.03358E-12	2.323340732			
Chst1	4.84666E-12	2.336577466			
Ltb	1.41622E-12	2.346021857			
Iapp	5.3603E-13	2.358109043			
Slc16a11	3.18256E-10	2.368407188			
Ccnb1	3.08399E-12	2.368904603			
Zc3h12a	2.67869E-11	2.38159798			
Mmp7	4.24497E-08	2.385877782			
Tmem173	3.85707E-12	2.394456178			
Mt2A	1.01806E-09	2.409524099			
Cxadrl1	3.29682E-12	2.415659703			
Cxcl3	4.68104E-11	2.417407498			
Ccl17	7.5222E-11	2.418288508			
C2cd4a	1.11528E-05	2.478766231			
Nusap1	3.29682E-12	2.525500088			
Ly6al	2.4163E-12	2.53239818			
Plk1	3.54602E-12	2.538130042			
C3	2.19665E-13	2.56258075			
Tnn	6.56337E-12	2.563201378			
Mab21l3	1.14885E-10	2.569238473			
Cx3cl1	1.55804E-12	2.581888387			
Ect2	5.37916E-10	2.59622488			
Fcnb	2.48968E-06	2.601743005			
Kif20a	2.86006E-11	2.610301265			
Birc5	1.23641E-13	2.630658002			

Dhrs9	2.70027E-10	1.883588898			
Duox1	4.65581E-08	1.884823592			
Mki67	4.01001E-17	1.889124154			
Mmp12	4.90538E-05	1.893903994			
Bpifa5	1.0331E-09	1.894348913			
Ube2t	4.25804E-09	1.894673599			
Depdc1	3.5539E-07	1.896444763			
Gch1	7.21523E-10	1.897083759			
Tac4	1.49793E-09	1.898150013			
Gabrp	4.86685E-13	1.898845977			
Mreg	7.67813E-13	1.898934073			
LOC103692892	1.25801E-06	1.902776013			
Adipoq	1.28763E-14	1.90342268			
Rspo2	1.12209E-08	1.9105805			
Tnn	2.41538E-09	1.922238025			
Kif22	4.87605E-13	1.925263773			
Tnfaip2	7.87956E-16	1.930123323			
Ccnb1	8.00229E-15	1.934723966			
Ttk	1.30324E-13	1.93903223			
LOC103691468	1.18249E-13	1.943574253			
Lilrb4	2.39723E-12	1.943655485			
Rrm2	3.63786E-14	1.945932626			
Sod2	1.66792E-17	1.949941376			
Rnf225	8.60643E-09	1.952882702			
Pclaf	2.51E-12	1.953420503			
Ccne1	1.9423E-13	1.954881374			
Nr4a3	1.9289E-12	1.958645658			
Hist1h1b	1.11911E-10	1.960167819			
Sapcd2	3.91315E-13	1.963062776			

Ly6i	2.19665E-13	2.643577982			
Rrm2	4.94315E-11	2.647588211			
Pclaf	7.98531E-13	2.66428077			
Ccna2	4.22703E-12	2.68708913			
RGD1309110	2.22544E-11	2.701425182			
S100a8	1.20494E-07	2.7499687			
Hist1h1b	2.55639E-10	2.756525519			
Chia	5.00531E-14	2.759757968			
Cep55	1.83797E-10	2.762334499			
Gsg1	2.61411E-08	2.793840885			
Slc16a6	8.76716E-15	2.871521017			
Sod2	1.12989E-14	2.88377886			
Tnfrsf9	5.48969E-13	2.899803247			
Shcbp1	4.94315E-11	2.905999033			
Kng1	8.96849E-11	2.944466173			
Knstrn	1.80795E-12	2.967875501			
Mmp12	2.357E-08	2.979418221			
Pttg1	3.95928E-12	2.980622498			
Hp	4.35582E-15	2.981137199			
Foxn4	2.15397E-11	2.989577498			
Kif22	9.17063E-12	2.990442228			
Tmem171	5.82013E-14	3.012090336			
Ube2c	3.08756E-13	3.012312985			
C4bpa	5.19451E-17	3.017029905			
Spc25	5.08348E-12	3.154101758			
Cxcl1	4.1733E-06	3.186897597			
Cenpw	1.5191E-10	3.234154532			
Cks2	1.00985E-12	3.255176658			
Aoc1	1.18709E-08	3.304806759			

Cd177	1.74711E-05	1.963756825			
Slamf8	4.08378E-14	1.969584363			
LOC102552128	3.24836E-10	1.977527931			
Serpine2	2.19315E-10	1.979641046			
Areg	1.17513E-06	1.981724642			
Cdh17	8.6861E-13	1.982738735			
Nusap1	2.15138E-19	1.999298364			
Mro	4.95199E-12	2.044067207			
Kif20a	5.37258E-13	2.046266323			
Iapp	2.18451E-12	2.051105975			
LOC102552017	1.45634E-07	2.06181424			
Plk1	4.95603E-16	2.064324088			
Kif11	9.86334E-13	2.065550333			
Degs2	3.51624E-11	2.071132974			
Hp	3.24981E-10	2.074217921			
Bcl2l14	4.86933E-14	2.100877318			
Pttg1	6.27695E-18	2.11389272			
LOC102548812	5.92866E-12	2.114590016			
Spc25	3.42204E-16	2.124313501			
Cdk1	3.99451E-15	2.132123285			
Ube2c	7.31509E-18	2.133095669			
A2m	5.91524E-06	2.157450915			
LOC102550929	6.33098E-09	2.163117368			
Kng2	3.68762E-09	2.164586994			
LOC689770	3.60998E-13	2.168848434			
Mab21l3	1.25782E-11	2.169428151			
Slc16a6	1.22448E-11	2.198973692			
C4bpa	1.41312E-09	2.203067976			
Cenpw	5.73308E-15	2.210150386			

Cdk1	1.00985E-12	3.345771668			
Fstl4	1.14509E-15	3.346351923			
Tnf	2.33409E-07	3.37193751			
S100a9	1.11104E-09	3.392969108			
Angptl1	4.35582E-15	3.400419287			
Cxcl6	1.5581E-10	3.414210956			
Itih1	5.32018E-17	3.430995747			
Cxcl2	9.35511E-05	3.513506541			
Tmc5	1.14509E-15	3.523625157			
Defb5	2.19665E-13	3.727264317			
Kng2	4.71665E-13	3.835509313			
Cdca3	8.5462E-13	3.887588994			
Chi3l1	4.07702E-17	3.962536226			
Cfi	5.64776E-16	3.999255616			
Ccl12	4.19176E-06	4.208395401			
Rhbdl2	2.30574E-13	4.239979301			
Lpo	9.63327E-11	4.365588603			
Ccl1	1.86957E-12	4.392721008			
Ccl7	3.11675E-09	4.669351754			
Noxo1	5.08348E-12	4.759283888			
Spp1	2.357E-08	4.864445142			
Muc3	5.82013E-14	4.96855864			
Cd177	7.42864E-14	5.780967846			
Ccl2	2.30785E-10	5.87042707			
Bpifb1	1.16607E-14	6.111117951			
Orm1	2.08744E-13	6.813357247			
Slc26a4	4.35582E-15	7.387151868			
Lcn2	7.37551E-14	8.963808461			
Prss30	2.21398E-18	9.799190767			

Il1r2	1.85424E-10	2.212835453			
LOC689757	1.06017E-13	2.237959805			
Klk1c12	4.86688E-09	2.245533818			
Arntl	9.12795E-08	2.254028153			
Knstrn	6.76036E-16	2.2639245			
Mt2A	1.80499E-10	2.274433783			
Traip	3.2444E-13	2.291315776			
Slamf9	2.29355E-11	2.297988777			
Fstl4	6.25154E-14	2.322123968			
Ly6i	4.24826E-10	2.348769845			
Cxcl2	1.15817E-08	2.369384836			
Ect2	2.29142E-15	2.41017091			
LOC102555481	8.48242E-12	2.413535861			
Selp	1.88675E-16	2.414366273			
Gsg1	1.08612E-07	2.428888978			
LOC102551752	2.85293E-13	2.43148634			
Inka1	1.36659E-09	2.457215833			
Vsig4	2.76982E-09	2.490535895			
Itih1	2.18356E-08	2.498112775			
S100a9	1.52837E-09	2.521619738			
Ocm2	2.62111E-05	2.549917078			
LOC102556856	4.33937E-12	2.557203914			
Tuba8	7.60249E-09	2.566585919			
Cenpq	4.18823E-12	2.593376504			
Chi3l1	1.17231E-09	2.599364211			
Rpl10l	1.33765E-06	2.609384267			
Cks2	3.10359E-17	2.613445383			
Angptl4	4.45947E-08	2.623189465			
Dock3	4.17337E-10	2.634726183			

Retnla	1.12989E-14	11.93707828			

LOC102550137	1.31894E-12	2.666868521			
Igh-6	2.09028E-07	2.683474735			
Cdca3	6.76405E-18	2.861344501			
Ccl1	3.14804E-11	2.866487618			
Sall4	8.80684E-09	2.871852257			
LOC691670	9.66634E-08	2.898205868			
Ccl12	4.34044E-11	2.920076241			
Scn10a	7.65761E-11	3.053333052			
Lcn2	3.58877E-08	3.065033525			
Lpo	2.37732E-08	3.079311916			
Klra2	1.1572E-05	3.128645577			
Chia	9.00899E-10	3.143864159			
Cfi	4.29497E-10	3.159336085			
Mmp7	2.85293E-13	3.161243865			
Imp3	9.14139E-11	3.189696931			
Ccl2	4.14945E-15	3.246886923			
Ly49i4	0.003877181	3.310803791			
RGD1309110	2.88508E-10	3.367237294			
Kng1	1.29147E-09	3.375908893			
Ly49s3	0.007324978	3.389888147			
Klk6	1.07085E-06	3.471244531			
LOC103691629	6.3558E-09	3.501168948			
LOC102551304	5.31781E-11	3.703487986			
Tmem104	7.22364E-11	4.116704795			
LOC102551003	2.90328E-10	4.179185162			
Aqp3	6.04362E-09	4.338389766			
Slc26a4	2.31033E-09	4.36119146			
Bpifb1	9.65039E-11	4.637785967			
Ccl7	1.28763E-14	4.712201905			

	LOC103691626	1.79524E-10	4.801546599			
	Prss30	2.59318E-18	4.987594059			
	Zfp18	5.75978E-11	5.970899287			
	LOC102549724	7.00812E-11	5.985801906			
	Spp1	3.43435E-10	8.30507598			
	Saal1	5.76423E-14	9.592156835			
	Orm1	9.74168E-15	10.58333174			
	Retnla	1.02301E-11	12.4064236			
	RGD1566373	6.026E-13	15.74064961			
	Tenm3	2.11602E-10	20.52358298			
	LOC102548289	2.95839E-10	29.95672406			
	Foxn4	7.13926E-12	56.31298967			
